# Consolidation and maintenance of long-term memory involve dual functions of the developmental regulator Apterous in clock neurons and mushroom bodies in the *Drosophila* brain

**DOI:** 10.1371/journal.pbio.3001459

**Published:** 2021-12-03

**Authors:** Show Inami, Tomohito Sato, Yuto Kurata, Yuki Suzuki, Toshihiro Kitamoto, Takaomi Sakai

**Affiliations:** 1 Department of Biological Sciences, Tokyo Metropolitan University, Tokyo, Japan; 2 Department of Anesthesia and Neuroscience & Pharmacology, Carver College of Medicine, University of Iowa, Iowa City, Iowa, United States of America; 3 Interdisciplinary Programs in Genetics and Neuroscience, University of Iowa, Iowa City, Iowa, United States of America; Washington University, St.Louis, MO 63110, UNITED STATES

## Abstract

Memory is initially labile but can be consolidated into stable long-term memory (LTM) that is stored in the brain for extended periods. Despite recent progress, the molecular and cellular mechanisms underlying the intriguing neurobiological processes of LTM remain incompletely understood. Using the *Drosophila* courtship conditioning assay as a memory paradigm, here, we show that the LIM homeodomain (LIM-HD) transcription factor Apterous (Ap), which is known to regulate various developmental events, is required for both the consolidation and maintenance of LTM. Interestingly, Ap is involved in these 2 memory processes through distinct mechanisms in different neuronal subsets in the adult brain. Ap and its cofactor Chip (Chi) are indispensable for LTM maintenance in the *Drosophila* memory center, the mushroom bodies (MBs). On the other hand, Ap plays a crucial role in memory consolidation in a Chi-independent manner in pigment dispersing factor (Pdf)-containing large ventral–lateral clock neurons (l-LNvs) that modulate behavioral arousal and sleep. Since disrupted neurotransmission and electrical silencing in clock neurons impair memory consolidation, Ap is suggested to contribute to the stabilization of memory by ensuring the excitability of l-LNvs. Indeed, ex vivo imaging revealed that a reduced function of Ap, but not Chi, results in exaggerated Cl^−^ responses to the inhibitory neurotransmitter gamma-aminobutyric acid (GABA) in l-LNvs, indicating that wild-type (WT) Ap maintains high l-LNv excitability by suppressing the GABA response. Consistently, enhancing the excitability of l-LNvs by knocking down GABA_A_ receptors compensates for the impaired memory consolidation in *ap* null mutants. Overall, our results revealed unique dual functions of the developmental regulator Ap for LTM consolidation in clock neurons and LTM maintenance in MBs.

## Abstract 2

A neurogenetic study using Drosophila reveals that the centrally expressed LIM-homeodomain transcription factor Apterous plays a crucial neuron-type-dependent role in two different memory processes - consolidation and maintenance of long-term memory.

## Introduction

A newly formed labile memory in the brain is consolidated into a more stable long-term memory (LTM), which is maintained until recall. The fruitfly *Drosophila melanogaster* is an excellent model organism for investigating the molecular mechanisms of learning and memory because it can efficiently learn and remember in various contexts, and advanced genetics is available for identifying and characterizing genes relevant to different aspects of memory processes [1–3]. Regardless of the learning paradigm, cAMP response element binding protein (CREB)-mediated transcription is essential for memory consolidation to establish LTM in *Drosophila* [4–6]. Many genes involved in memory consolidation have been identified, and its underlying molecular mechanisms are becoming elucidated [7,8]. LTM maintenance also requires the transcriptional activation of CREB in *Drosophila* [9,10], suggesting that de novo protein synthesis via transcription is also necessary for LTM maintenance. Mushroom bodies (MBs), a higher-order brain center for learning and memory [1–3], play critical roles in both the consolidation and maintenance of LTM [9,10]. However, despite recent progress, the molecular and cellular mechanisms underlying the consolidation and maintenance of LTM remain incompletely understood.

Previously, we and others reported the unexpected finding that the *Drosophila* circadian clock gene *period* (*per*) plays a vital role in memory consolidation induced by courtship conditioning and aversive olfactory classical conditioning [4,11,12]. This finding suggests that *per*-expressing clock neurons are also essential for LTM. In *Drosophila*, clock neurons expressing the neuropeptide pigment dispersing factor (Pdf; hereafter referred to as Pdf neurons) are critically involved in generating circadian rhythms. Pdf neurons consist of 2 neural clusters, small ventral–lateral neurons (s-LNvs) and large ventral–lateral neurons (l-LNvs) [13]. s-LNvs are believed to act as circadian pacemaker neurons [13,14], and l-LNvs are essential for sleep/wake regulation [15,16]. We have recently found that Pdf in l-LNvs also regulates the maintenance of LTM induced by courtship conditioning (hereafter referred to as courtship LTM) [9]. Thus, intercellular communication from l-LNvs to MB neurons is likely to play a crucial role in courtship LTM maintenance. However, little is known about whether Pdf neurons are also involved in other memory processes (e.g., consolidation and recall of LTM) and how Pdf neurons modulate courtship LTM formed and maintained in MBs.

*Drosophila* Apterous (Ap) is one of the most well-studied LIM homeodomain (LIM-HD) proteins. Similar to other LIM-HD proteins, Ap has 2 protein–protein interaction domains (LIM) and a DNA-binding homeodomain motif (HD). Ap coordinates with its cofactor Chip (Chi), and the multimeric Ap/Chi complex (hereafter referred to as Ap/Chi) acts as a positive regulator of transcription [17]. In *Drosophila*, *ap* was initially identified as a regulatory gene in wing development [18,19]. The subsequent investigations revealed that Ap and Chi are also essential for nervous system development [17,20–23]. Since Ap continues to be expressed in many neurons in the adult fly brain, it may have brain functions other than the regulation of neural development. In fact, Ap in the adult brain neurons expressing a neuropeptide Pdf is required for proper sleep/wake regulation [24]. Furthermore, Aranha and colleagues have reported that *ap* is expressed in MB neurons using an *ap* enhancer trap GAL4 line previously shown to accurately report *ap* expression [25]. Therefore, Ap in the MBs may play a role in *Drosophila* learning and memory. However, functions of Ap in MBs remain unclarified.

In this study, using a courtship conditioning assay, we examined if and how Ap in the adult brain is involved in *Drosophila* learning and memory. In the courtship conditioning assay, male flies are paired with unreceptive mated females, which give sufficient stresses (e.g., courtship-inhibiting cues and sexual rejection) to males to interfere with mating success (conditioning). After being conditioned, their memory is subsequently observed as the suppression of male courtship even toward virgin females. Previous studies have identified several genes involved in memory consolidation to establish courtship LTM. These include (1) *Cyclic-AMP response element-binding protein B* (*CrebB*) encoding a transcriptional factor [4]; (2) *Notch* encoding a transmembrane receptor [26]; (3) *orb2* encoding an mRNA-binding protein [27]; and (4) *Ecdysone Receptor* encoding a nuclear hormone receptor [28]. Since these gene products regulate protein expression, it is most likely that de novo protein synthesis is essential for memory consolidation to establish courtship LTM. CrebB and Notch are also essential for *Drosophila* LTM induced by olfactory associative learning [4–6,29]. Thus, molecular mechanisms of de novo protein synthesis for memory consolidation may be shared by different learning paradigms. Similarly to the consolidation of courtship LTM, its maintenance also requires CrebB-mediated transcription [9]. However, the molecular mechanisms of courtship LTM maintenance still remain largely elusive.

Here, we provide evidence indicating that Ap and Chi in MBs are essential for maintaining courtship LTM, whereas Ap in Pdf neurons is necessary for memory consolidation through the regulation of the appropriate Cl^−^ responses to gamma-aminobutyric acid (GABA) in a Chi-independent manner.

## Results

### *ap* mutants are defective in LTM

**Fig 1:**
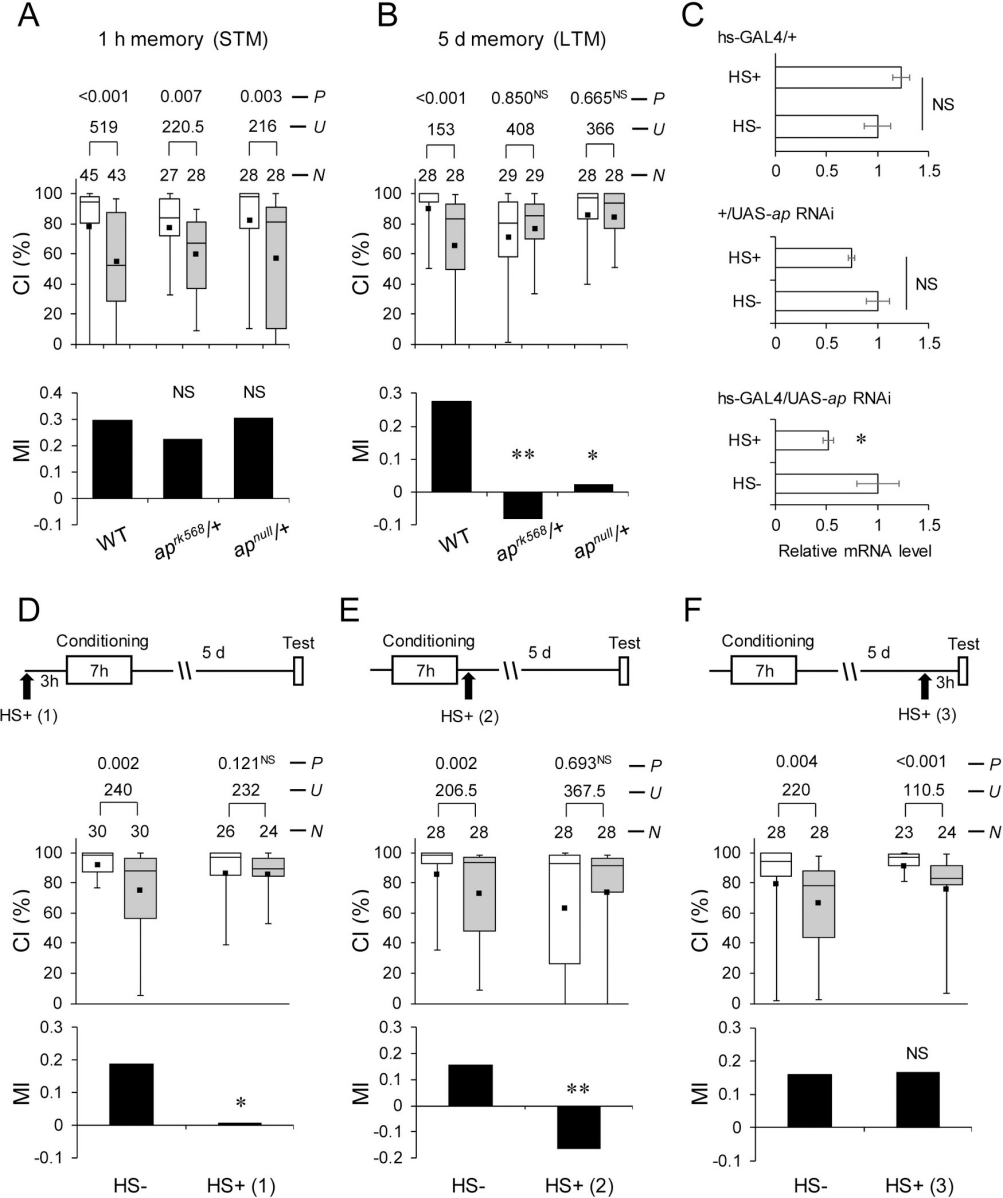
Ap is necessary for LTM. The underlying data can be found in S1 Data. **(A)** WT, *ap*^*rk568*^/+, and *ap*^*null*^/+ flies were used in the experiments. Males were tested for 1 hour after 1-hour conditioning (1-hour memory). **(B)** WT, *ap*^*rk568*^/+, and *ap*^*null*^/+ flies were used in the experiments. Males were tested on day 5 after 7-hour conditioning (5-day memory). **(C)** Real-time qRT-PCR analysis of *ap* mRNA expression level. hs-GAL4 was used for the induction of *ap* RNAi. Mean ± SEM was calculated from 5 to 6 replicates. HS−, non–heat-shocked flies. HS+, flies with heat-shock treatment (20 minutes) 3 hours before RNA extraction. *, *P* < 0.05; NS, not significant. Error bars show SEM in each figure. **(D)** Five-day memory after 7-hour conditioning in flies with or without a 20-minute heat-shock treatment 3 hours before conditioning [HS+ (1)]. **(E)** Five-day memory after 7-hour conditioning. HS+ (2), flies with heat-shock treatment immediately after conditioning. **(F)** Five-day memory after 7-hour conditioning. HS+ (3), flies with heat-shock treatment 3 hours before test. (A, B, and D–F) Box plots for a set of CI data show fifth, 25th, 75th, and 95th centiles. In the box plots, the black square in each box indicates the mean, the line in each box is drawn at the median, the white boxes indicate naive males, and the gray boxes indicate conditioned males. CI, courtship index; MI, memory index; *N*, sample size; *U*, Mann–Whitney *U*; *P*, probability; *, *P* < 0.05; **, *P* < 0.01; NS, not significant. (D–F), hs-GAL4/UAS-*ap* RNAi flies were used. Ap, Apterous; LTM, long-term memory; qRT-PCR, quantitative reverse transcription PCR; RNAi, RNA interference; WT, wild-type.

We examined whether Ap is involved in *Drosophila* memory using a courtship conditioning assay. In this assay, 1-hour conditioning generates short-term memory (STM), and 7-hour conditioning induces LTM [4,30,31]. STM persists for at least 1 hour, whereas LTM persists for at least 5 days [4,31]. Two mutant alleles of *ap* (*ap*^*rk568*^ and *ap*^*UGO35*^) were tested for the effects of *ap* mutations on LTM. *ap*^*rk568*^ is a strong hypomorphic allele with a P element insertion into the putative 5′-flanking region [18]. *ap*^*UGO35*^ (hereafter referred to as *ap*^*null*^) is a null allele generated by the imprecise excision of the P element associated with *ap*^*rk568*^ [18]. Since *ap*^*rk568*^ and *ap*^*null*^ are homozygous lethal, we used heterozygous mutants (*ap*^*rk568*^/+ and *ap*^*null*^/+). In wild-type (WT), *ap*^*rk568*^/+, and *ap*^*null*^/+ flies, the courtship index (CI), an indicator of male courtship activity, of conditioned males was significantly lower than that of naive males 1 hour after 1-hour conditioning (Fig 1A, CI). To quantify courtship memory, memory index (MI) was calculated (refer to Materials and Methods). No significant differences in MI were detected among 3 genotypes (Fig 1A, MI; WT versus*ap*^*rk568*^/+, Permutation test; *P* = 0.550; WT versus *ap*^*null*^/+, Permutation test; *P* = 0.896). Thus, in *ap*^*rk568*^/+ and *ap*^*null*^/+, 1-hour memory was intact. Furthermore, we examined whether male courtship suppression is detected 8 hours and 24 hours after 1-hour conditioning. The courtship suppression of conditioned males was evident in WT and *ap*^*rk568*^/+ flies 8 hours after 1-hour conditioning (S1 Fig), whereas it was not detected 24 hours after 1-hour conditioning. These results demonstrate that courtship STM is maintained for at least 8 hours and that the ability of heterozygous *ap*^*rk568*^ flies to learn, form, and maintain STM is comparable to that of WT flies.

On day 5 after 7-hour conditioning (hereafter referred to as 5-day memory), WT flies also showed a significant reduction in CI in a conditioning-dependent manner (Fig 1B, CI). However, no significant differences in CI were detected between naive and conditioned *ap*^*rk568*^/+ and *ap*^*null*^/+ (Fig 1B, CI), and their LTM was impaired (Fig 1B, MI; WT versus *ap*^*rk568*^/+, Permutation test; *P* = 0.004; WT versus *ap*^*null*^/+, Permutation test; *P* = 0.015), indicating that Ap is essential for LTM specifically.

### Ap expression during the adult stage is necessary for LTM

Since Ap plays essential roles in neurodevelopmental events in the central nervous system [17], it is possible that the observed LTM impairment is caused by neurodevelopmental defects in *ap* mutant flies. To determine if the *ap* expression in adulthood is critical for LTM, we examined the effects of temporal knockdown of *ap* expression on the LTM phenotype using flies heterozygous for heat-shock (hs)-GAL4 and UAS-*ap* RNA interference (RNAi) lines (hs-GAL4/UAS-*ap* RNAi). First, the effectiveness of *ap* RNAi was confirmed by quantitative reverse transcription PCR (qRT-PCR). Flies were heat-shocked (37°C) for 20 minutes, and total RNA was extracted 3 hours after heat-shock treatment. Heat-shocked hs-GAL4/UAS-*ap* RNAi flies showed about 50% reduction in *ap* expression level compared with control flies (Fig 1C; hs-GAL4/+, Student *t* test, *t*_(8)_ = −1.474, *P* = 2.306; +/UAS-*ap* RNAi, *U* = 5, *P* = 0.117; hs-GAL4/UAS-*ap* RNAi, *U* = 4, *P* = 0.025). The *ap* expression level recovered to the non–heat-shocked level 48 hours after heat-shock treatment (S2 Fig). In the absence of heat-shock treatment, LTM in hs-GAL4/UAS-*ap* RNAi flies was detected [Fig 1D–1F; CI in HS− (1), *U* = 240, *P* = 0.002; CI in HS− (2), *U* = 206.5, *P* = 0.002; CI in HS− (3), *U* = 220, *P* = 0.004]. By contrast, LTM impairment was observed when flies were heat-shocked 3 hours before or immediately after 7-hour conditioning (Fig 1D and 1E; HS− versus HS+(1), Permutation test; *P* = 0.037; HS− versus HS+(2), Permutation test; *P* = 0.007). The impairment is due to the conditional knockdown of *ap* because heat-shock treatment did not affect LTM in GAL4 and UAS control flies (hs-GAL4/+ and UAS-*ap* RNAi/+ flies; S3 Fig). These results suggest that normal Ap function is required for the consolidation and maintenance of LTM. However, when flies were heat-shocked 3 hours before the test, LTM was intact (Fig 1F; HS−(3) versus HS+(3), Permutation test; *P* = 0.956). On the basis of the results of the qRT-PCR analysis (Fig 1C), the *ap* expression level must be reduced to approximately 50% of the control level during the test. Therefore, reduced Ap function does not have a critical effect on LTM retrieval. Under this condition, the timing of the initiation of heat-shock treatment is considered to be within the memory maintenance phase. However, the treatment did not affect LTM. Therefore, the reduction in Ap expression level and the duration of reduced Ap expression (≤3 hours) induced by the treatment may be insufficient to inhibit the LTM maintenance.

### Ap in MB α/β neurons is indispensable for memory maintenance

**Fig 2:**
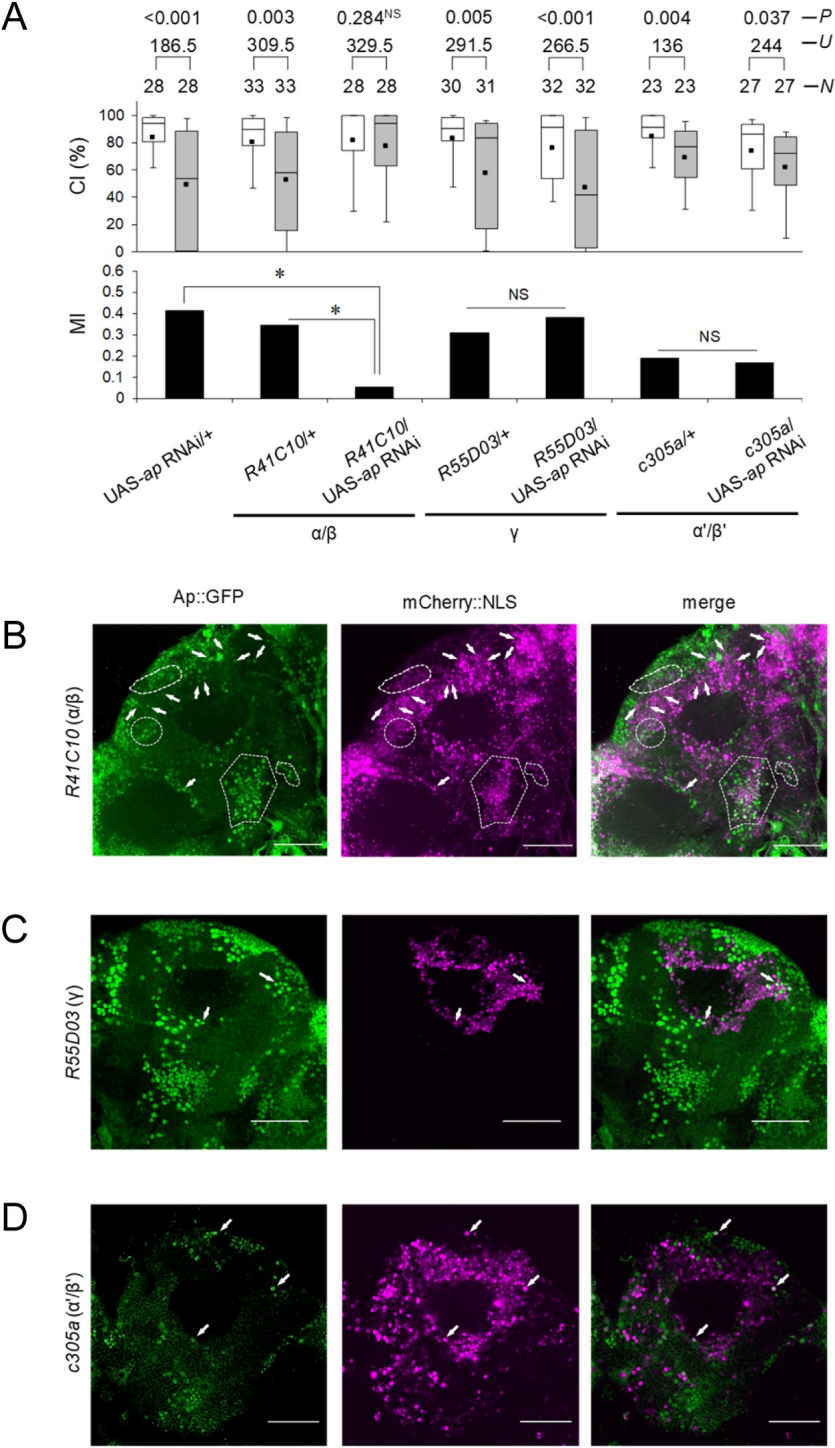
*ap* knockdown in MB α/β neurons induces LTM impairment. **(A)** Five-day memory after 7-hour conditioning. *ap* RNAi was driven by MB α/β -GAL4 (*R41C10*), MB γ-GAL4 (*R55D03*), and MB α’/β’-GAL4 (*c305a*). Box plots for a set of CI data show 10th, 25th, 75th, and 90th centiles. In the box plots, the black square in each box indicates the mean, the line in each box is drawn at the median, the white boxes indicate naive males, and the gray boxes indicate conditioned males. The underlying data can be found in S1 Data. CI, courtship index; MI, memory index; *N*, sample size; *U*, Mann–Whitney *U*; *P*, probability; *, *P* < 0.05; NS, not significant. **(B–D)** Ap-expressing neurons in MBs. Confocal sectional images at the level of Kenyon cells in MBs. Scale bars, 50 μm; green, Ap::GFP; magenta, mCherry::NLS driven by MB-GAL4 lines. Dotted lines and arrows show the colocalization of Ap::GFP and mCherry::NLS. (B) *ap*::*GFP*/+; UAS-*mCherry*::*NLS*/*R41C10* flies were used. (C) *ap*::*GFP*/*+*; UAS-*mCherry*::*NLS*/*R55D03* flies were used. (D) *ap*::*GFP*/*c305a*; UAS-*mCherry*::*NLS*/*+* flies were used. Ap, Apterous; LTM, long-term memory; MB, mushroom body; RNAi, RNA interference.

To examine the significance of Ap in the nervous system for memory consolidation and/or maintenance, we knocked down *ap* in neurons using UAS-*ap* RNAi in combination with several GAL4 lines. When a pan-neural *nSyb*-GAL4 line was used, 2 independent UAS-*ap* RNAi transgenes (NIG-fly and TRiP) both resulted in impaired LTM (S4A Fig), indicating that Ap in the nervous system is essential for LTM. In the following experiments, UAS-*ap* RNAi (NIG-fly) was used. To examine whether Ap in the *Drosophila* memory center is involved in LTM, we used 2 MB-GAL4 lines, *OK107* and *30Y*. In both lines, GAL4 is expressed in all MB lobes [32]. *ap* knockdown using these MB-GAL4 lines impaired LTM (S5B Fig). Furthermore, to define the particular MB neurons critical for courtship LTM, 3 GAL4 lines (*R41C10*, *R55D03*, and *c305a*) driving GAL4 expression in different neuronal subsets of MBs were used [9]. LTM was intact when Ap was knocked down in MB γ or α’/β’ neurons, whereas Ap knockdown in MB α/β neurons impaired LTM (Fig 2A, MI; *R41C10*/+ versus *R41C10* / UAS-*ap* RNAi, Permutation test, *P* = 0.011; UAS-*ap* RNAi/+ versus *R41C10* / UAS-*ap* RNAi, Permutation test; *P* = 0.033). In *R41C10*, GAL4 expression is controlled by a 3,500-bp fragment from the intronic region of *ap*. Both Jenelia’s Flylight project (https://flweb.janelia.org/cgi-bin/view_flew_imagery.cgi?line=R41C10) and our previous study [9] demonstrated that the reporter gene expression driven by *R41C10* is primarily observed in MB αβ neurons. Although Jenelia’s Flylight project detected signals in optic lobes and visual projection neurons, the expression outside the MB αβ neurons is very weak and limited. Thus, the result with *R41C10* indicates that MB αβ neurons are most likely responsible for Ap-dependent courtship LTM.

We next examined whether Ap-expressing neurons are present in MBs. To visualize Ap-expressing neurons, we first used an *ap* mutant allele *ap*^*md544*^ with a P-element (pGawB) insertion (hereafter referred to as *ap*^*GAL4*^). *ap*^*GAL4*^ was previously shown to accurately report the expression of *ap* [33]. Flies homozygous for *ap*^*GAL4*^ were lethal, but the heterozygous flies were viable [20]. In *ap*^*GAL4*^/UAS-*mCD8*::*GFP* flies, GFP signals, which colocalized with Fas II, a marker of MB lobes, were primarily detected in a subset of MB αβ neurons but not in MB γ neurons or α’/β’ neurons (S6A Fig). Furthermore, they were abolished by MB-GAL80 (S6B Fig), indicating that *ap*^*GAL4*^ signals indeed came from the MB neurons. Next, we used *ap*::*GFP* knock-in flies, which express a GFP reporter in a pattern consistent with endogenous Ap expression [34]. Ap::GFP and the nucleus-targeted mCherry reporter for MB α/β neurons were colocalized in many neurons (Fig 2B), and only a few neurons showed colocalization in MB γ or α’/β’ neurons (Fig 2C and 2D). Combined, these results support the conclusion that Ap is predominantly expressed in MB αβ neurons.

To clarify whether *ap* mutations induce structural defects in the MB, we used *ap*^*GAL4*^ flies. Flies homozygous for *ap*^*GAL4*^ were lethal, as was previously reported [20]. However, we were able to obtaine escapers of the F_1_ hybrid of *ap*^*GAL4*^ and *ap*^*rk568*^ flies (*ap*^*GAL4*^/*ap*^*rk568*^) in which the *ap* function is severely reduced. We confirmed that they exhibit obvious structural defects of the MB with the α lobes branched into 2 regions (S7A Fig, arrows). By contrast, no such structural defects of the MB were identified in *ap*^*GAL4*^/*+* (S6A and S7B Figs). These results are consistent with the previous report indicating that escapers of the F_1_ hybrid of *ap*^*GAL4*^ and *ap*^*P44*^ (a null allele) show severe morphological defects such as abnormal wings and an embryonic VNC structure, but that *ap*^*GAL4*^/+ flies show no such abnormalities [20]. Furthermore, in *ap*^*GAL4*^/+ flies as well as *ap*^*null*^/+ and *ap*^*rk568*^/+ flies, 1-hour memory after 1-hour conditioning (STM) was intact, whereas 5-day memory after 7-hour conditioning (LTM) was defective (S6C and S6D Fig). In summary, flies heterozygous for *ap* mutant alleles did not show obvious structural defects in the MB, and STM was intact. Nonetheless, they showed LTM impairment.

**Fig 3:**
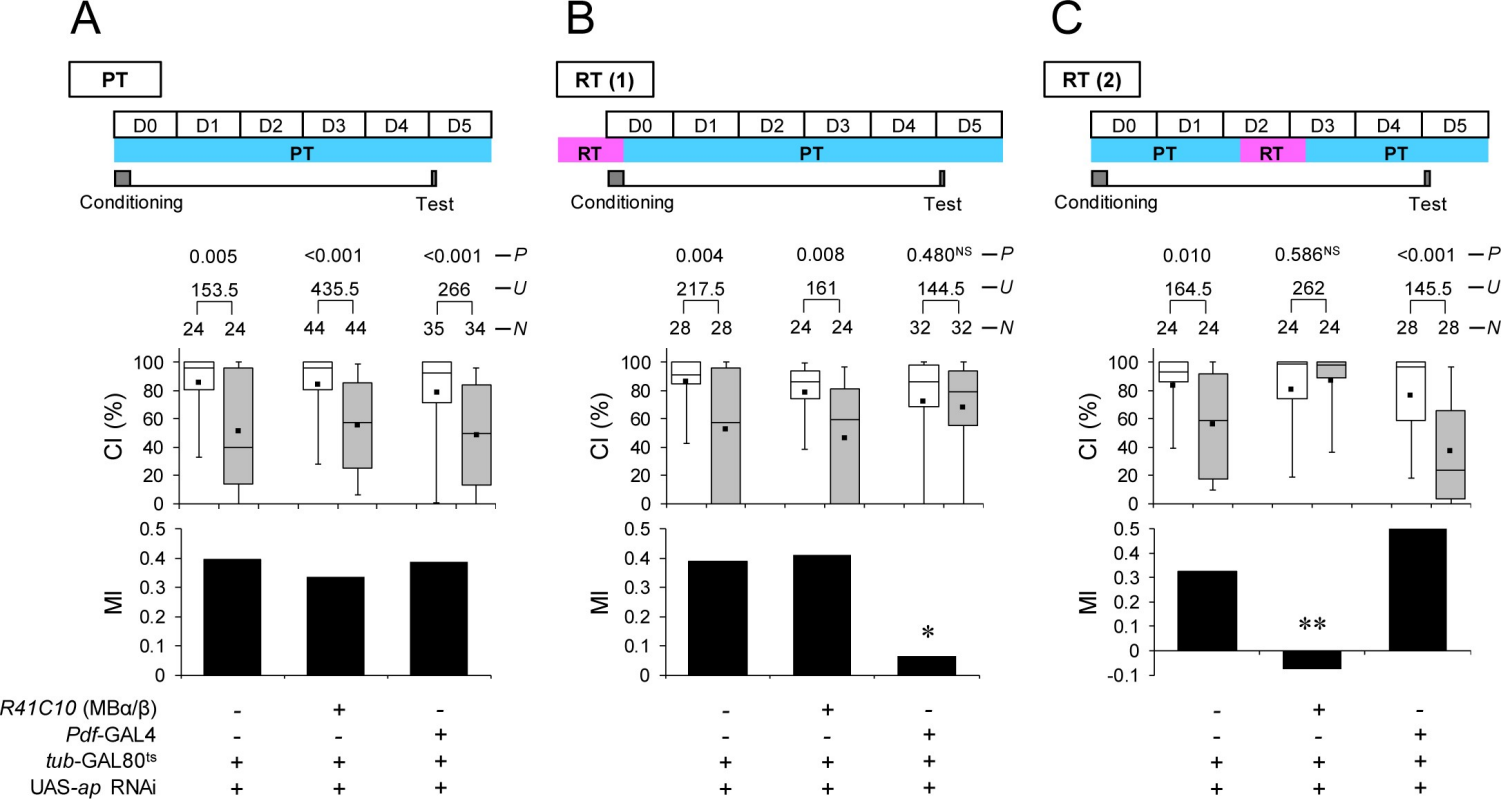
Temporary *ap* knockdown in MB α/β or Pdf neurons induces LTM impairment. **(A)** All experiments were performed at PT (25°C). **(B)** Flies were kept at RT for 24 hours before the end of conditioning. **(C)** Flies were kept at RT for 48 to 72 hours after 7-hour conditioning. (A–C) Memory on day 5 in UAS-*ap* RNAi/+; *tub*-GAL80^ts^/*R41C10* and control (UAS-*ap* RNAi/*tub*-GAL80^ts^) flies. Box plots for a set of CI data show fifth, 25th, 75th, and 95th centiles. In the box plots, the black square in each box indicates the mean, the line in each box is drawn at the median, the white boxes indicate naive males, and the gray boxes indicate conditioned males. The underlying data can be found in S1 Data. CI, courtship index; MI, memory index; *N*, sample size; *U*, Mann–Whitney *U*; *P*, probability; *, *P* < 0.05; **, *P* < 0.01; NS, not significant. Ap, Apterous; LTM, long-term memory; MB, mushroom body; Pdf, pigment dispersing factor; PT, permissive temperature; RNAi, RNA interference; RT, restrictive temperature.

To further explore whether Ap in MB α/β neurons is responsible for memory consolidation or maintenance, we temporarily knocked down *ap* in MB α/β neurons using the TARGET system [35]. To knock down *ap* during the memory consolidation or maintenance phase, the temperature was increased to 30°C for 24 hours during 2 different experimental periods: (1) starting at 24 hours before the end of conditioning (conditioning phase) [Fig 3B, restrictive temperature (RT) (1)]; and (2) starting at 48 hours after the end of conditioning (memory maintenance phase) [Fig 3C, RT(2)]. When flies were kept at a permissive temperature (PT), they showed normal LTM regardless of their genotypes (Fig 3A). On the other hand, LTM was impaired when Ap was knocked down during the memory maintenance phase (Fig 3C), although it was intact when Ap was knocked down during the conditioning phase (Fig 3B). Thus, Ap in MB α/β neurons mainly contributes to LTM maintenance rather than memory consolidation.

### Ap in Pdf neurons is indispensable for memory consolidation

**Fig 4:**
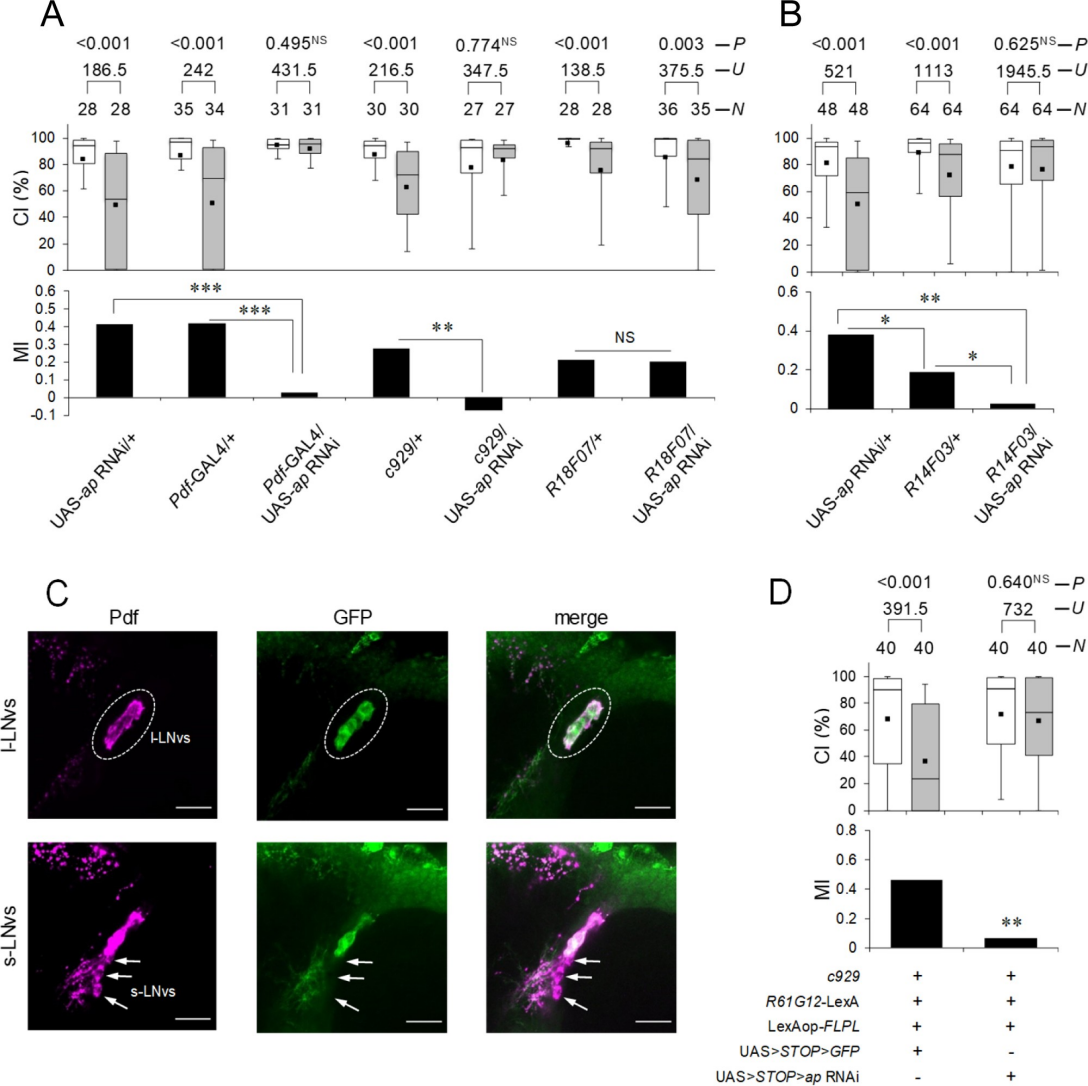
*ap* knockdown in Pdf-positive l-LNvs induces LTM impairment. **(A)** Five-day memory after 7-hour conditioning. *ap* RNAi was driven by *Pdf*-GAL4, *c929*, and *R18F07*. Box plots for a set of CI data show 10th, 25th, 75th, and 90th centiles. In the box plots, the black square in each box indicates the mean, the line in each box is drawn at the median, the white boxes indicate naive males, and the gray boxes indicate conditioned males. CI, courtship index; MI, memory index; *N*, sample size; *U*, Mann–Whitney *U*; *P*, probability; **, *P* < 0.01; ***, *P* < 0.001; NS, not significant. **(B)** Five-day memory after 7-hour conditioning. *ap* RNAi was driven by *R14F03*. *, *P* < 0.05; **, *P* < 0.01; NS, not significant. **(C)** l-LNv–specific expression of mCD8::GFP. *c929*/*R61G12*-LexA; LexAop-*FLPL*/UAS>STOP>*mCD8*::*GFP* flies were used. Brains were stained with an anti-Pdf antibody and an anti-GFP antibody. Magenta, Pdf; green, mCD8::GFP. Confocal section images at the level of Pdf neurons of the adult brain. Scale bars represent 10 μm. **(D)** l-LNv–specific knockdown of *ap*. Five-day memory after 7-hour conditioning. *c929*/*R61G12*-LexA; LexAop-*FLPL*/UAS>STOP>*ap* RNAi flies were used. **, *P* < 0.01; NS, not significant. (B and D) Box plots for a set of CI data show fifth, 25th, 75th, and 95th centiles. In the box plots, the black square in each box indicates the mean, the line in each box is drawn at the median, the white boxes indicate naive males, and the gray boxes indicate conditioned males. CI, courtship index; MI, memory index; *N*, sample size; *U*, Mann–Whitney *U*; *P*, probability. (A, B, and D). The underlying data can be found in S1 Data. Ap, Apterous; l-LNv, large ventral–lateral clock neuron; LTM, long-term memory; Pdf, pigment dispersing factor; RNAi, RNA interference.

Ap knockdown by hs-GAL4 shows that Ap is involved in both memory consolidation and maintenance (Fig 1). Since Ap in MB α/β neurons mainly contributed to LTM maintenance but not to consolidation (Fig 3C), it is likely that brain neurons other than MB α/β neurons are involved in Ap-dependent memory consolidation. We previously identified that Ap is expressed in Pdf neurons consisting of s-LNvs and l-LNvs [24]. Thus, we next examined whether *ap* knockdown in Pdf neurons affects LTM. First, the following 3 GAL4 lines were used: (1) *Pdf*-GAL4, which drives GAL4 expression in both s-LNvs and l-LNvs; (2) *c929*, which drives GAL4 expression in peptidergic neurons including l-LNvs [36]; and (3) *R18F07*, which drives GAL4 expression in s-LNvs and only weakly in one of the l-LNvs [9]. When *Pdf*-GAL4 and *c929* were used to knock down *ap*, LTM was impaired (Fig 4A; Permutation test; in *Pdf*-GAL4/UAS-*ap* RNAi, versus UAS control, *P* < 0.001, versus GAL4 control, *P* < 0.001; in *c929*/UAS-*ap* RNAi, versus UAS control, *P* < 0.001, versus GAL4 control, *P* = 0.001), whereas *ap* knockdown by *R18F07* did not impair LTM (Fig 4A; in *R18F07*/UAS-*ap* RNAi, versus UAS control, *P* = 0.099, versus GAL4 control, *P* = 0.916). These results suggest that Ap in l-LNvs is necessary for LTM. Since GAL4 expression in *c929* is detected in the neuronal subset of the abdominal ganglion [37] and Pdf neuropeptide is also expressed in the abdominal ganglion [38], Ap in the abdominal ganglion may be necessary for LTM. Thus, we next used a GAL4 line, *R14F03*, which drives GAL4 expression in l-LNvs but not in the abdominal ganglion (https://flweb.janelia.org/cgi-bin/view_flew_imagery.cgi?line=R14F03). If Ap in the abdominal ganglion is essential for LTM, *ap* knockdown using *R14F03* should not affect LTM. However, *R14F03*/UAS-*ap* RNAi flies showed LTM impairment (Fig 4B), indicating that Ap in l-LNvs is necessary for LTM. Next, to determine whether Ap in l-LNvs but not in other brain neurons is essential for LTM, we performed l-LNv–specific Ap knockdown using the Flp-out system [39] with *c929* and *R61G12*-LexA. In *R61G12*-LexA, LexA is expressed in l-LNvs and s-LNvs [9]. Using this system, we can knock down targeted genes specifically in the *c929* and *R61G12*-LexA-coexpressing neurons. The effectiveness of the Flp-out system was confirmed using the GFP reporter. In control flies carrying c929, *R61G12*-LexA, LexAop-*FLPL*, and UAS>STOP> *GFP*, the GFP signal was detected in all l-LNvs but not in s-LNvs (Fig 4C). As shown in Fig 4D, the Flip-out experiments with UAS>STOP>*ap* RNAi demonstrated that l-LNv–specific *ap* knockdown impaired LTM (Permutation test, *P* = 0.002). This result also supports the conclusion that Ap in l-LNvs is indispensable for LTM.

Next, we examined when *ap* in Pdf neurons is required during processing for LTM. In contrast to temporal knockdown in MB α/β neurons, LTM was impaired when Ap was knocked down during conditioning but not during maintenance (Fig 3B; Permutation test; *P* = 0.019). These findings indicate that Ap in Pdf neurons mainly contributes to memory consolidation rather than LTM maintenance.

### *ap* expression in MB α/β and Pdf neurons rescues LTM phenotype in heterozygous *ap*^*null*^ flies

**Fig 5:**
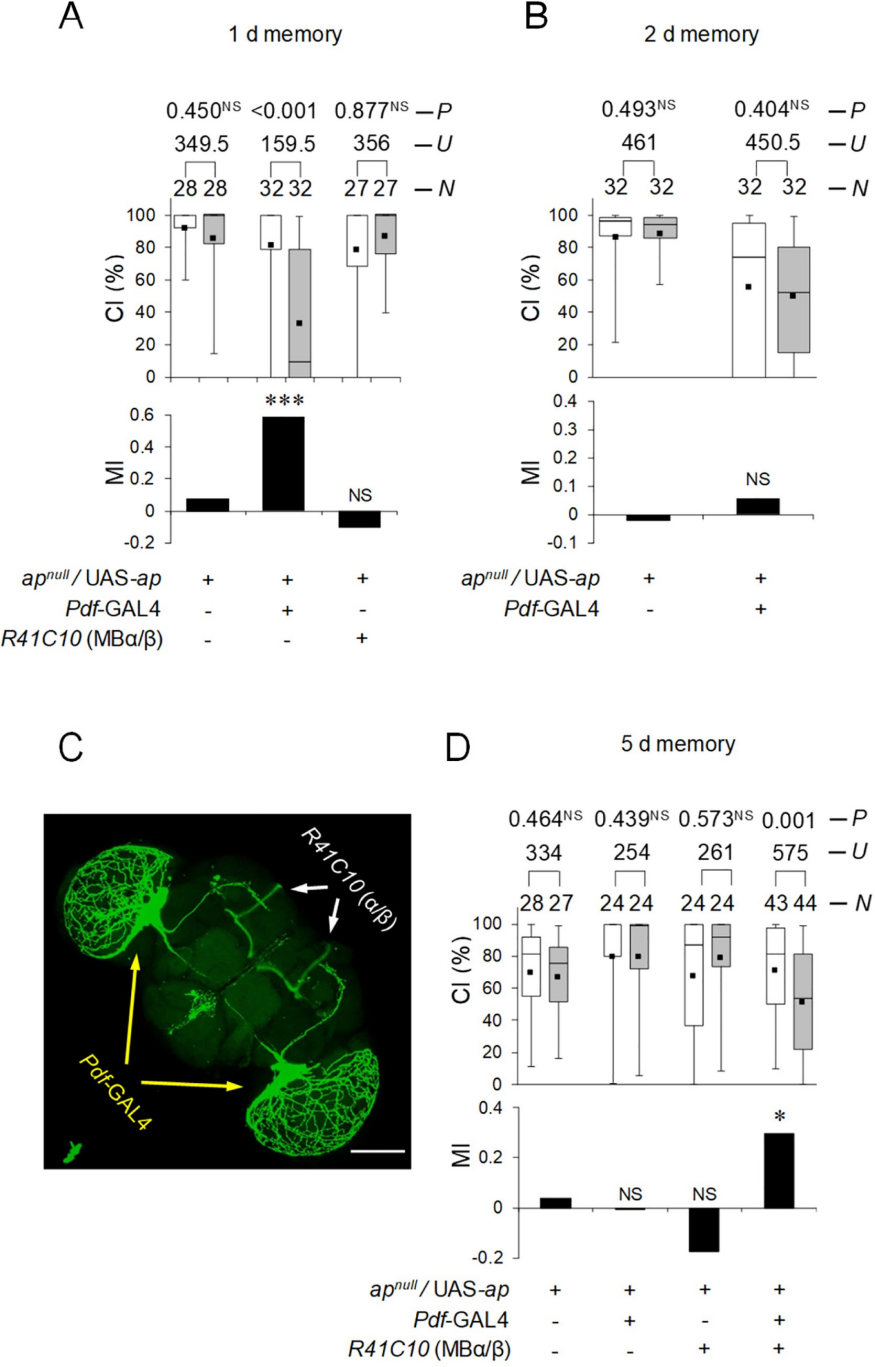
*ap* expression in MB α/β and Pdf neurons in *ap*^*null*^/+ flies restores LTM. **(A, B, and D)** *ap*^*null*^/UAS-*ap* flies were used as a control. Box plots for a set of CI data show 5th, 25th, 75th, and 95th centiles. In the box plots, the black square in each box indicates the mean, the line in each box is drawn at the median, the white boxes indicate naive males, and the gray boxes indicate conditioned males. The underlying data can be found in S1 Data. CI, courtship index; MI, memory index; *N*, sample size; *U*, Mann–Whitney *U*; *P*, probability; *, *P* < 0.05; ***, *P* < 0.001; NS, not significant. (A) One-day memory after 7-hour conditioning. (B) Two-day memory after 7-hour conditioning. (C) Stacked confocal image showing an anterior view of the adult brain. The scale bar represents 100 μm. *Pdf*-GAL4/UAS-*mCD8*::*GFP*; *R41C10*/+ flies were used. (D) Five-day memory after 7-hour conditioning. Ap, Apterous; LTM, long-term memory; MB, mushroom body; Pdf, pigment dispersing factor.

Since cell type–specific *ap* knockdown experiments revealed that sufficient *ap* function is required for normal LTM, we next examined whether *ap* expression in MB α/β and/or Pdf neurons can rescues the LTM phenotype in *ap*^*null*^/+ flies. If 1-day memory impairment after 7-hour conditioning in *ap*^*null*^/+ flies is due to defective memory consolidation resulting from the reduced function of Ap in Pdf neurons, the ability of *ap*^*null*^/+ flies to show 1-day memory should be restored by expressing WT *ap* in Pdf neurons. As shown in Fig 5A, the targeted expression of *ap* in Pdf neurons rescued the impairment of 1-day memory in *ap*^*null*^/+ flies (Fig 5A; Permutation test; *P* < 0.001). However, the targeted expression of *ap* in MB α/β neurons did not rescue the impairment of 1-day memory in *ap*^*null*^/+ flies (Fig 5A; Permutation test; *P* = 0.130). Furthermore, Pdf neuron–specific *ap* expression did not rescue impairment of memory on day 2 after 7-hour conditioning in *ap*^*null*^/+ flies (Fig 5B, 2-day memory; Permutation test, *P* = 0.496). These findings indicate that Pdf neuron–specific Ap expression in *ap*^*null*^/+ flies is insufficient to maintain LTM for 2 days. For 5-day memory, the targeted expression of *ap* in either Pdf or MB α/β neurons was not sufficient to rescue the *ap*^*null*^/+ phenotype (Fig 5D; Permutation test, versus *Pdf*-GAL4, *P* = 0.778, versus *R41C10*, *P* = 0.259). However, when *ap* was expressed in both Pdf and MB α/β neurons using *Pdf*-GAL4; *R41C10* flies (Fig 5C), LTM impairment in *ap*^*null*^/+ flies was rescued (Fig 5D; Permutation test; *P* = 0.042). These findings support the idea that Ap in Pdf neurons is responsible for memory consolidation, whereas that in MB α/β neurons is responsible for LTM maintenance.

### Chi in MB α/β neurons, but not in Pdf neurons, is necessary for LTM

**Fig 6:**
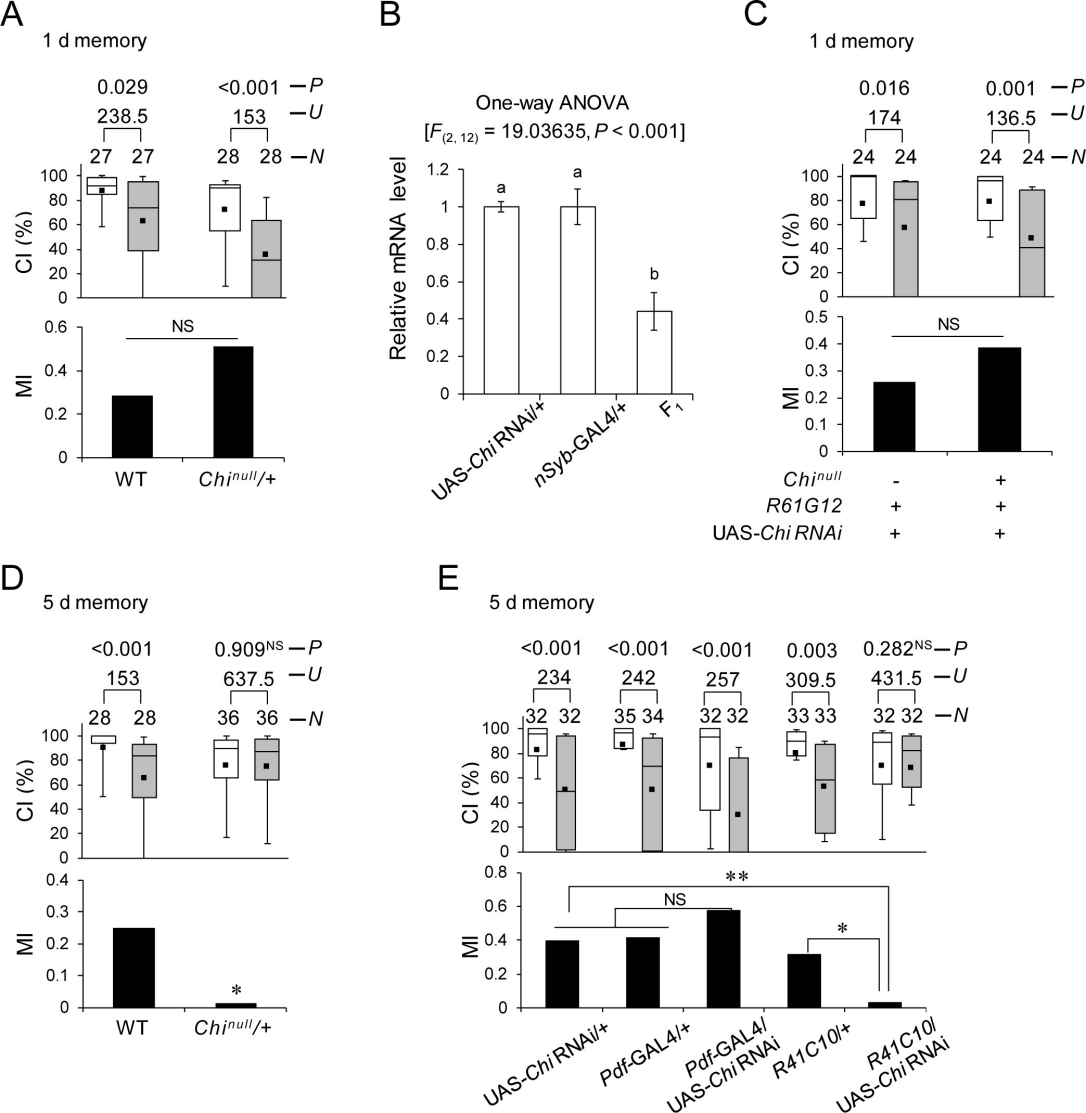
*Chi* in MB α/β is necessary for LTM. The underlying data can be found in S1 Data. **(A)** One-day memory after 7-hour conditioning. WT and *Chi*^*null*^/+ flies were used. NS, not significant. **(B)** Real-time qRT-PCR analysis of *ap* mRNA expression level. *nSyb*-GAL4 was used for the induction of *Chi* RNAi (VDRC). Mean ± SEM was calculated from 5 replicates. One-way ANOVA followed by post hoc analysis using Scheffe test for multiple comparisons was used. Bars with the same letter indicate values that are not significantly different (*P* > 0.05). **(C)** Pdf neuron–specific *Chi* knockdown in *Chi*^*null*^/+ flies. One-day memory after 7-hour conditioning. UAS-*Chi RNAi/*+; *R61G12/+* (control) and UAS-*Chi RNAi/Chi*^*null*^; *R61G12/+* flies were used. **(D)** Five-day memory after 7-hour conditioning. WT and *Chi*^*null*^/+ flies were used. *, *P* < 0.05. (A, C, and D) Box plots for a set of CI data show fifth, 25th, 75th, and 95th centiles. In the box plots, the black square in each box indicates the mean, the line in each box is drawn at the median, the white boxes indicate naive males, and the gray boxes indicate conditioned males. CI, courtship index; MI, memory index; *N*, sample size; *U*, Mann–Whitney *U*; *P*, probability. **(E)** *Chi* knockdown using *Pdf*-GAL4 and *R41C10*. Five-day memory after 7-hour conditioning. Box plots for a set of CI data show 10th, 25th, 75th, and 90th centiles. In the box plots, the black square in each box indicates the mean, the line in each box is drawn at the median, the white boxes indicate naive males, and the gray boxes indicate conditioned males. CI, courtship index; MI, memory index; *N*, sample size; *U*, Mann–Whitney *U*; *P*, probability; *, *P* < 0.05; **, *P* < 0.01; NS, not significant. Chi, Chip; LTM, long-term memory; MB, mushroom body; qRT-PCR, quantitative reverse transcription PCR; RNAi, RNA interference; WT, wild-type.

Since the transcriptional activity of Ap requires the formation of a protein complex with a cofactor, Chi [23,40], we reasoned that Chi is also involved in LTM. To investigate this possibility, a null allele *Chi*^*e5*.*5*^ (hereafter referred to as *Chi*^*null*^) was used [41]. Since *Chi*^*null*^ is also homozygous lethal as was observed in *ap*^*null*^, we used heterozygous mutant flies (*Chi*^*null*^/+). Unlike the LTM phenotype in *ap*^*null*^/+, 1-day memory after 7-hour conditioning was not impaired in *Chi*^*null*^/+ flies (Fig 6A; Permutation test, *P* = 0.087). Since a 50% reduction of *Chi* function in *Chi*^*null*^/+ flies may be insufficient to induce LTM impairment, we further knocked down *Chi* in Pdf neurons in *Chi*^*null*^/+ flies and examined the effect of this knockdown on LTM. The effectiveness of *Chi* RNAi (VDRC) was confirmed by qRT-PCR using a pan-neuronal GAL4 line (*nSyb*-GAL4); *Chi* RNA levels were reduced by about 55% (Fig 6B; Scheffe’s multiple comparisons, GAL4 control versus F_1_, *P* < 0.001, UAS control versus F_1_, *P* < 0.001). Even when *Chi* was knocked down in a Pdf neuron–specific manner in *Chi*^*null*^/+ background flies, 1-day memory was not affected (Fig 6C; Permutation test, *P* = 0.488). In contrast to 1-day memory, 5-day memory in *Chi*^*null*^/+ flies was impaired (Fig 6D, Permutation test, *P* = 0.026), indicating that Chi is involved in LTM maintenance rather than in memory consolidation.

**Fig 7:**
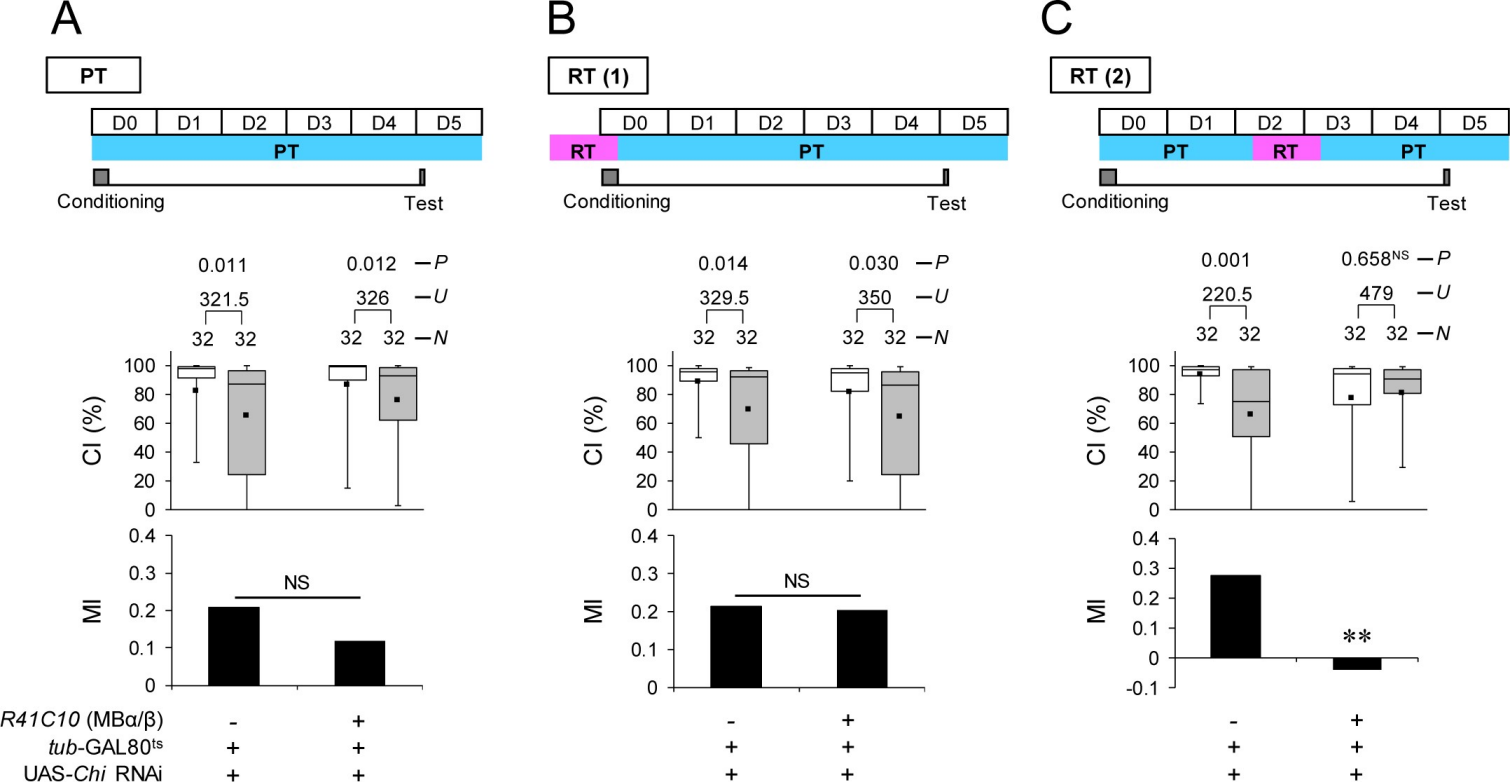
Temporary *Chi* knockdown in MB α/β neurons induces LTM impairment. **(A)** All experiments were performed at PT (25°C). **(B)** Flies were kept at RT for 24 hours before the end of conditioning. **(C)** Flies were kept at RT for 48 to 72 hours after 7-hour conditioning. (A–C) Five-day memory after 7-hour conditioning. UAS-*Chi* RNAi (VDRC)/+; *tub*-GAL80^ts^/*R41C10* and control (UAS-*Chi* RNAi (VDRC)/*tub*-GAL80^ts^) flies were used. Box plots for a set of CI data show fifth, 25th, 75th, and 95th centiles. In the box plots, the black square in each box indicates the mean, the line in each box is drawn at the median, the white boxes indicate naive males, and the gray boxes indicate conditioned males. The underlying data can be found in S1 Data. CI, courtship index; MI, memory index; *N*, sample size; *U*, Mann–Whitney *U*; *P*, probability; **, *P* < 0.01; NS, not significant. Chi, Chip; LTM, long-term memory; MB, mushroom body; PT, permissive temperature; RNAi, RNA interference; RT, restrictive temperature.

To verify that Chi is required in neurons for 5-day memory, *nSyb*-GAL4/UAS-*Chi* RNAi (VDRC) and *nSyb*-GAL4/UAS-*Chi* RNAi (TRiP) flies were used. We found that the pan-neuronal knockdown of *Chi* with 2 independent UAS-*Chi* RNAi lines both impaired 5-day memory (S4B Fig). In the following experiments, UAS-*Chi* RNAi (VDRC) was used. We confirmed that *Chi* knockdown in MB α/β neurons induces 5-day memory impairment (Fig 6E; Permutation test, *R41C10*/UAS-*Chi* RNAi versus UAS control, *P* = 0.007, *R41C10*/UAS-*Chi* RNAi versus GAL4 control, *P* = 0.015). However, *Chi* knockdown in Pdf neurons had no effect on LTM (Fig 6E). Next, we temporarily knocked down *Chi* in MB α/β neurons to examine when *Chi* is required in these neurons during memory processing for LTM. LTM was impaired when *Chi* was knocked down during the memory maintenance phase but not during the conditioning phase (Fig 7C; Permutation test; *P* = 0.002). Thus, we concluded that Chi in MB α/β neurons contributes to memory maintenance rather than LTM consolidation. Taken together, these findings support the idea that Ap cooperates with Chi in MB α/β neurons to maintain LTM, but plays a role in l-LNvs to consolidate memory independently of Chi.

### Disruption of neurotransmission and electrical silencing of Pdf neurons impairs memory consolidation

**Fig 8:**
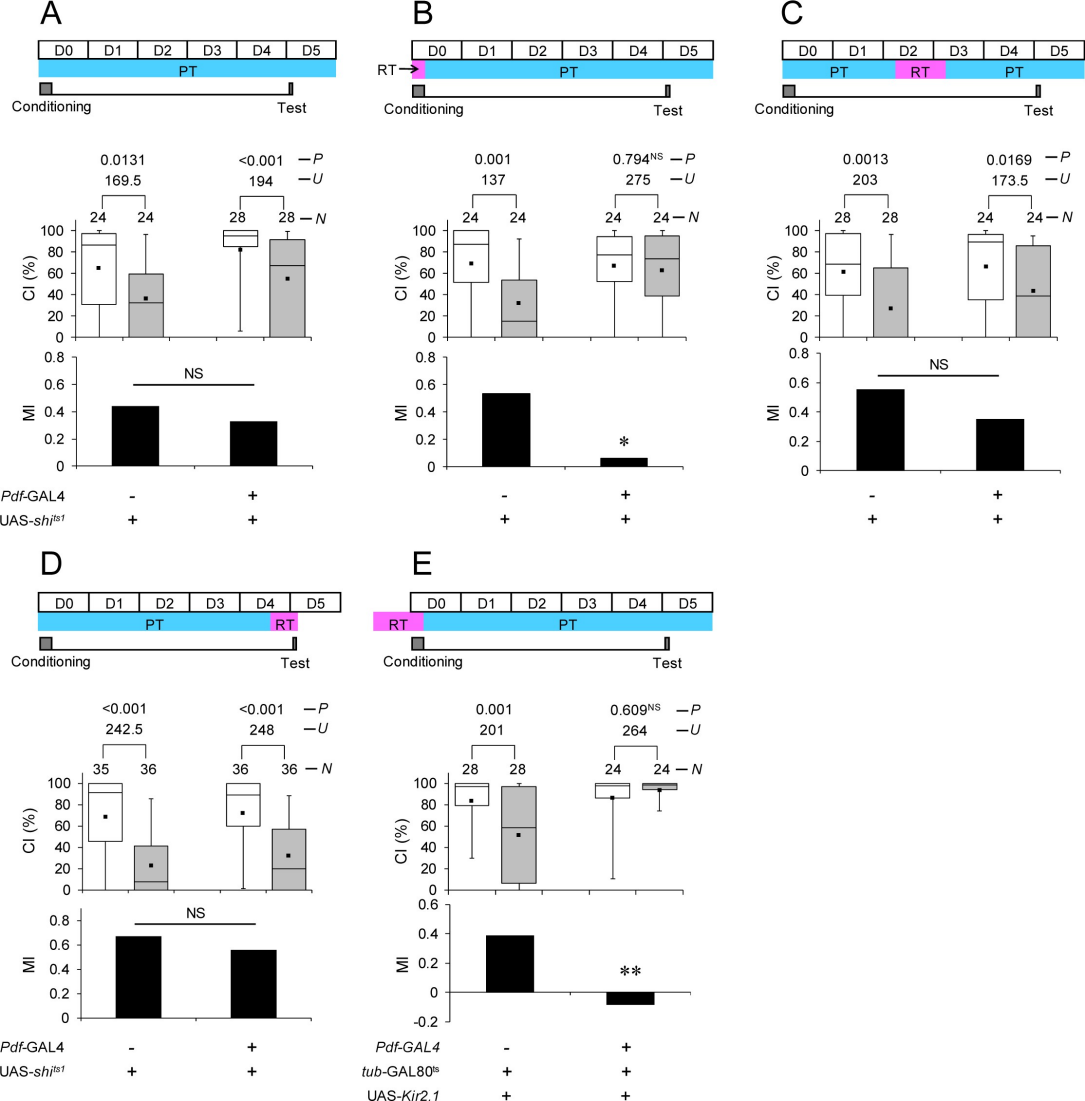
The disruption of the neurotransmission and electrical silencing of Pdf neurons impairs memory consolidation. **(A–D)** Five-day memory after 7-hour conditioning. *Pdf*-GAL4/+ (control) and *Pdf*-GAL4/UAS-*shi*^*ts1*^ flies were used. *, *P* < 0.05; **, *P* < 0.01; NS, not significant. (A) All experiments were performed at PT (25°C). (B) Flies were kept at RT during conditioning. (C) Flies were kept at RT for 48 to 72 hours after 7-hour conditioning. (D) Flies were kept at RT for 12 hours before the test. (E) Five-day memory after 7-hour conditioning. Flies were kept at RT for 24 hours before the end of conditioning. UAS-*Kir2*.*1*/*Pdf*-GAL4; *tub*-GAL80^ts^/+ flies were used. UAS-*Kir2*.*1*/*tub*-GAL80^ts^ flies were used as the control. (A–E) Box plots for a set of CI data show fifth, 25th, 75th, and 95th centiles. In the box plots, the black square in each box indicates the mean, the line in each box is drawn at the median, the white boxes indicate naive males, and the gray boxes indicate conditioned males. The underlying data can be found in S1 Data. CI, courtship index; MI, memory index; *N*, sample size; *U*, Mann–Whitney *U*; *P*, probability. Pdf, pigment dispersing factor; PT, permissive temperature; RT, restrictive temperature.

To determine whether neurotransmission from Pdf neurons is necessary for proper memory consolidation, we used the temperature-sensitive Dynamin mutation s*hibire*^*ts1*^ (*shi*^*ts1*^). The targeted expression of *shi*^*ts1*^ can inhibit neurotransmission in a spatially specific and temperature-dependent manner [42]. *Pdf*-GAL4/UAS-*shi*^*ts1*^ flies showed LTM at PT (Fig 8A). We found that the disruption of neurotransmission during the conditioning phase impaired LTM (Fig 8B), but not during the memory maintenance or test phase (Fig 8C and 8D). Thus, neurotransmission from Pdf neurons is necessary for proper memory consolidation. In addition, we performed the electrical silencing of Pdf neurons using the inwardly rectifying Kir2.1 channel combined with the TARGET system. For the silencing of Pdf neurons during memory consolidation, we shifted the temperature from PT to RT and vice versa during the conditioning phase. As was observed in the disruption of neurotransmission using *shi*^*ts1*^, the electrical silencing of Pdf neurons during conditioning impaired LTM (Fig 8E).

### Ap in Pdf neurons is indispensable for appropriate response to GABA

Our results show that Ap in Pdf neurons is necessary for memory consolidation in a Chi-independent manner (Figs 3, 5, and 6). Since both the disruption of neurotransmission and the electrical silencing mimic the effect of Ap loss of function and impair memory consolidation, *ap* mutations or knockdown may reduce the excitability of Pdf neurons. A *Drosophila* ionotropic GABA_A_ receptor (GABA_A_R), Resistant to Dieldrin (Rdl), is expressed in Pdf neurons. Anti-RDL antibody staining reveals that RDL is highly expressed in l-LNv somata, while little or no expression is observed in s-LNv somata [15, 43]. Furthermore, electrophysiological analysis revealed that l-LNvs respond to GABA [43]. Considering the physiological properties of l-LNvs, we next investigated the possibility that Ap in l-LNvs is involved in the response to GABA.

**Fig 9:**
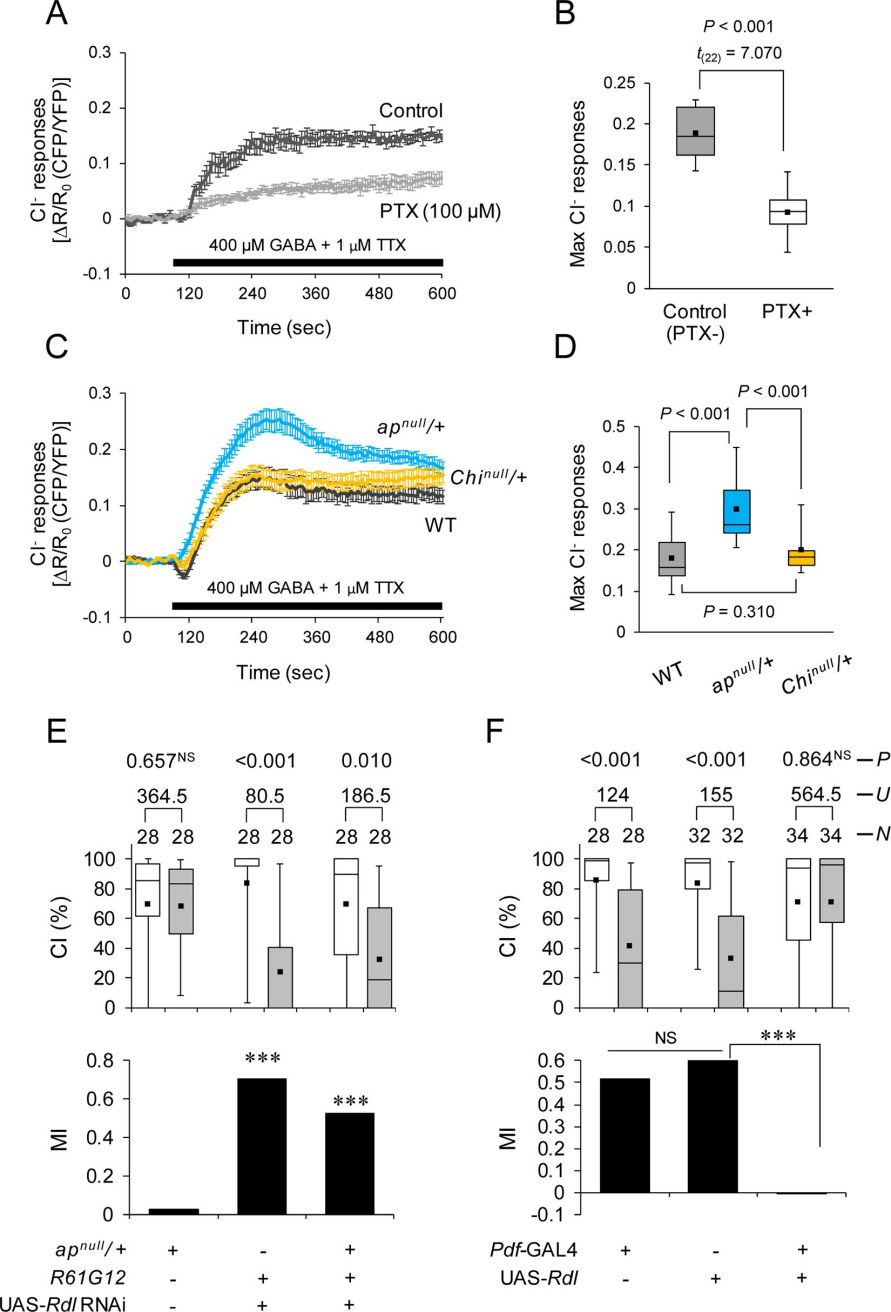
Ap in l-LNvs is indispensable for appropriate response to GABA. The underlying data can be found in S1 Data. **(A–D)** Ex vivo imaging using SuperClomeleon. (A and B) UAS-*SuperClomeleon*/*R61G12* flies were used. PTX, picrotoxin. (A) Traces of mean SuperClomeleon response to 400 μM GABA and 1 μM TTX with or without bath-applied 100 μM PTX. Error bars, SE; *N* = 12 in each trace. (B) Maximum percentage change in fluorescence of SuperClomeleon related to (A). Student *t* test was used. (C) UAS-*SuperClomeleon*/*R61G12* flies (WT), UAS-*SuperClomeleon*/*ap*^*null*^; *R61G12*/+ (*ap*^*nill*^/+), and UAS-*SuperClomeleon*/*Chi*^*null*^; *R61G12*/+ (*Chi*^*null*^/+) flies were used. Traces of mean SuperClomeleon response to 400 μM GABA and 1 μM TTX. Error bars, SE; *N* = 20 to 24 in each trace. (D) Maximum percentage change in fluorescence of SuperClomeleon related to (C). Nonparametric ANOVA (Kruskal–Wallis test) followed by post hoc analysis using the Steel–Dwass test was carried out for multiple comparisons. **(E and F)** One-day memory after 7-hour conditioning. ***, *P* < 0.001; NS, not significant. (E) *Rdl* knockdown in Pdf neurons in *ap*^*null*^/+. *ap*^*null*^/+, UAS-*Rdl* RNAi/*R61G12*, and *ap*^*null*^/UAS-*Rdl* RNAi; *R61G12* flies were used. (F) *Rdl* overexpression in Pdf neurons. *Pdf*-GAL4/+, UAS-*Rdl/+*, and *Pdf*-GAL4/UAS-*Rdl* flies were used. (B, D, E, and F) Box plots show fifth, 25th, 75th, and 95th centiles. In the box plots, the black square in each box indicates the mean, the line in each box is drawn at the median. Ap, Apterous; GABA, gamma-aminobutyric acid; l-LNv, large ventral–lateral clock neuron; Pdf, pigment dispersing factor; RNAi, RNA interference; WT, wild-type.

To visualize the response to GABA in l-LNvs, the FRET-based Cl^−^ probe, SuperClomeleon, was used [44,45]. First, in flies with WT *ap*, we confirmed robust Cl^−^ responses to 400 μM GABA in l-LNvs in the presence of TTX, while the responses were blocked by the GABA_A_R antagonist picrotoxin (Fig 9A and 9B). Similar Cl^−^ responses were observed in heterozygous *Chi*^*null*^ flies (Fig 9C and 9D). Compared with WT and heterozygous *Chi*^*null*^ flies, heterozygous *ap*^*null*^ flies showed robust increases in Cl^−^ responses (Fig 9C and 9D), indicating that the response to GABA in l-LNvs is augmented when the *ap* function is suppressed. Since Rdl is also expressed in MB neurons [46], we investigated whether Ap in MB α/β neurons is also involved in the response to GABA. As was observed in l-LNvs, Cl^−^ responses in MB α lobes in heterozygous *ap*^*null*^ flies, but not in heterozygous *Chi*^*null*^ flies, were greater than those in WT flies (S8A and S8B Fig). These results indicate that Ap is necessary for appropriate Cl^−^ responses to GABA in a Chi-independent manner.

In Pdf neurons, *ap* knockdown impaired 1-day memory after 7-hour conditioning (Fig 4), while *Chi* knockdown did not (Fig 6E). Similarly, increased responses to GABA in l-LNvs were detected in *ap*^*null*^/+ but not in *Chi*^*null*^/+. Thus, the overresponses to GABA in l-LNvs may cause 1-day memory impairment in *ap*^*null*^/+ flies. If the reduced Ap expression in *ap*^*null*^/+ leads to a decrease in l-LNv excitability due to augmented responses to the inhibitory neurotransmitter GABA and the exaggerated inhibition of LNv excitability causes the 1-day memory impairment, the 1-day memory phenotype in heterozygous *ap*^*null*^ flies may be compensated for by the reduced *Rdl* expression in l-LNvs. As we expected, the knockdown of *Rdl* in Pdf neurons compensated for the impaired 1-day memory in *ap*^*null*^/+ flies (Fig 9E). Furthermore, the Pdf neuron–specific overexpression of *Rdl* in WT background induced 1-day memory impairment (Fig 9F). This result is consistent with the aversive effect of the electrical silencing of Pdf neurons on LTM (Fig 8).

Since increased responses to GABA in MB α lobes were also detected in *ap*^*null*^/+ (S8 Fig), it is possible that the overresponse to GABA in MB α/β neurons is the cause of the impairment of 5-day memory in *ap*^*null*^/+ flies. However, unlike the Pdf neurons, the overexpression of *Rdl* in MB α/β neurons driven by *R41C10* did not affect 5-day memory (S8C Fig).

## Discussion

The evolutionarily conserved LIM-HD protein Ap acts as a transcriptional activator, and it is essential for various developmental events in *Drosophila* [18,19,21,23]. However, the functions of Ap in mature adults have remained largely unknown. In this study, we found that Ap in the l-LNvs and MB α/β neurons in the adult brain plays an acute physiological role in the consolidation and maintenance of LTM induced by courtship conditioning. This conclusion was drawn mainly from the observation that the neuronal subset-specific knockdown of *ap* in adult flies results in the impairment of specific memory processes.

Although Ap is critical for normal development [17], including the development of the nervous system, we do not consider that the LTM defects in flies with *ap* loss of function are caused by developmental failure because no obvious structural defects in MB were identified in *ap*^*GAL4*^/*+* (S6A Fig) and *ap*^*null*^/+ (S7B Fig) flies. Furthermore, we previously reported that Pdf neuron–specific *ap* knockdown does not affect the number of Pdf-positive cells and varicosities [24]. Thus, heterozygous *ap* mutant or *ap* knockdown flies are unlikely to have significant developmental effects on the brain structure. On the other hand, we cannot completely rule out the possibility that a 50% reduction in Ap function induces minor developmental defects in brain neurons (e.g., abnormal neuronal projection and synaptogenesis). However, although the *ap* expression of MB neurons should be about 50% of that of the WT flies in *ap*^*null*^/*Pdf*-GAL4; UAS-*ap*/+ flies, 1-day memory after 7-hour conditioning was intact in these flies (Fig 5). Therefore, even if the heterozygous *ap*^*null*^ mutation causes minor developmental defects in neural projection and synaptogenesis in MB neurons, these flies can consolidate memory and maintain it for 1 day. Furthermore, in the experiments on the temporal knockdown of *ap*, we revealed that adult-specific *ap* expression is essential for courtship memory consolidation and maintenance (Fig 3). Taken together, we concluded that centrally expressed Ap is critical for the acute physiological regulation of the consolidation and maintenance of courtship LTM.

**Fig 10:**
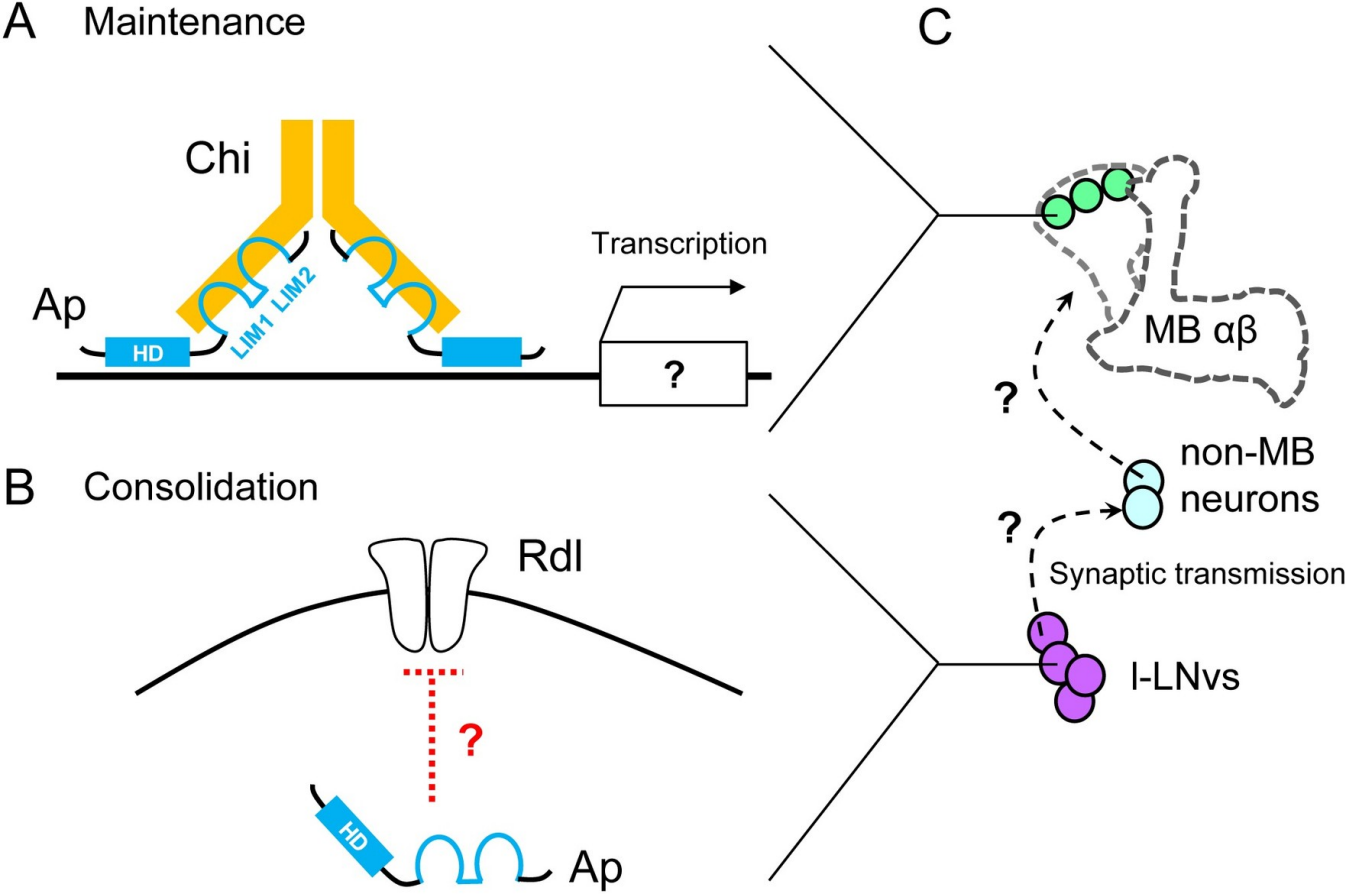
Possible model of Ap functions in the consolidation and maintenance of LTM. **(A)** The Ap/Chi complex induces gene expression. Subsequently, proteins required for maintaining consolidated LTM are produced in MB α/β neurons. **(B)** In l-LNvs, Ap may inhibit GABA responses via Rdl in a Chi-independent manner. **(C)** Intercellular communication from l-LNvs to MB neurons plays a crucial role in forming stable LTM. Non-MB neurons may mediate intercellular communication between l-LNvs and MB neurons. Ap, Apterous; Chi, Chip; GABA, gamma-aminobutyric acid; l-LNv, large ventral–lateral clock neuron; LTM, long-term memory; MB, mushroom body.

We identified that Ap and its cofactor Chi in MB α/β neurons are indispensable for LTM maintenance. Ap LIM domains interact with the Chi LIM interaction domain, and Chi can homodimerize through the dimerization domain [17,23,40] (Fig 10A). Since Ap/Chi regulates the transcription of Ap target genes [17], it is most likely that Ap/Chi is necessary to alter gene expression profiles in MB α/β neurons so that proteins required for the maintenance of courtship LTM are appropriately provided at the correct time and place (Fig 10A). It is important to identify the changes in gene expression in MB α/β neurons that are dependent on courtship conditioning, and are critical for LTM maintenance. Future experiments using cell type–specific transcriptome analysis will help us address this important issue.

In *Drosophila* wing development, it has been proposed that Beadex (Bx) [also known as *Drosophila* LIM-only protein (dLMO)] modulates the Ap function by interfering with the formation of Ap/Chi [47]. In this scenario, Bx can modify Ap-dependent transcription in the presence of Chi in the adult fly brain. In fact, *Bx* expression is prominent in several distinct regions of the adult brain including MBs and Pdf neurons [48]. Since the Ap/Chi complex is critical for LTM maintenance in MB α/β neurons, Bx may be involved in LTM maintenance by regulating the Ap/Chi complex activity. Interestingly, Bx in MB neurons is involved in the LTM maintenance induced by aversive olfactory conditioning [10], implying that the Ap/Chi complex plays an important role in LTM in different learning paradigms. It will be interesting to see whether Bx also affects the maintenance of courtship LTM via the modification of Ap/Chi-dependent transcription in MB α/β neurons.

Unlike the Chi-dependent role of Ap in MB α/β neurons, Ap in Pdf-positive l-LNvs was essential for memory consolidation to establish courtship LTM in a Chi-independent manner. Thus, the molecular mechanisms responsible for the Ap function in l-LNvs should be different from those in the previously reported model proposing that Ap forms a complex with Chi and regulates the transcription. For the Chi-independent Ap function, we provide evidence that Ap is necessary for appropriate Cl^−^ responses to GABA in l-LNvs; reduced *ap* function enhanced the Cl^−^ responses in l-LNvs. By contrast, reduced *Chi* function did not affect the Cl^−^ response to GABA. Considering the Ap mutant phenotype, WT Ap likely contributes to reducing the responses of l-LNv to GABA and maintains their higher excitability. Consistent with the effect of the *ap* mutation on LTM consolidation and responses to GABA in l-LNv, we found that *Rdl* knockdown in Pdf neurons compensates for the defective 1-day memory observed in heterozygous *ap* null mutant flies. These results indicate that the *ap*-dependent and *Chi*-independent regulation of responses to GABA in l-LNv is critical for LTM consolidation. Although it remains elusive how Ap regulates the responses to GABA, our findings raise the possibility that Ap reduces Cl^−^ influx by suppressing the expression of Rdl in l-LNvs or modulating the channel properties (e.g., receptor trafficking and localization) (Fig 10B). Further study will be needed to examine the factors that modify the responses to GABA owing to *ap* mutations in l-LNvs.

As was observed in l-LNvs (Fig 9), Ap also regulates responses to GABA in MB α lobes (S8 Fig), which are the critical neuronal subsets for Ap-dependent LTM maintenance. However, the modulation of GABA responses in MB neurons may not be essential for LTM maintenance because *Rdl* overexpression in MB α/β neurons has no effect on LTM (S8C Fig), although it induces LTM impairment in Pdf neurons. Furthermore, the knockdown of *Chi* in MB α/β neurons has no effect on GABA responses in MB α lobes (Fig 9) but severely disrupts LTM maintenance (Fig 7). Taken together, in *R41C10*-positive MB α/β neurons, the transcriptional activity of Ap/Chi rather than the modulation of GABA responses is considered to be involved in LTM maintenance.

As is well known in the olfactory associative learning paradigm, MB neurons are critically involved in memory consolidation to establish courtship LTM [9,28,31,49,50]. In this study, we identified that synaptic transmission from l-LNvs is necessary for memory consolidation (Fig 8), suggesting that intercellular communication from l-LNvs to MB neurons plays a crucial role in establishing stable LTM. Since the l-LNvs arborize in the most distal layer of the medulla and the accessory medullae (aMe), and they project to the other hemisphere through the posterior optic tract (POT) [51], it is unlikely that l-LNvs synaptically project and transmit to MB α/β neurons directly. Although the details of the specific neural circuits from l-LNvs to MBs are still unknown, non-MB interneurons likely mediate communication between l-LNvs and MB neurons (Fig 10C). The identification of neuronal circuits connecting l-LNvs and MB neurons will be one of the important subjects of our future studies.

Sleep is essential for the consolidation of *Drosophila* courtship memory [12,52]. In addition, Pdf-positive l-LNvs regulate light-dependent arousal [16], and knockdown of Ap and Chi in l-LNvs reduces the sleep amount [24]. These findings suggest that reduced sleep caused by Ap knockdown impairs memory consolidation. However, this is unlikely the case because *Chi* knockdown in Pdf neurons did not affect 1-day memory (Fig 6), even though it reduces the sleep amount [24]. Thus, the impairment of memory consolidation induced by Ap knockdown in l-LNvs does not merely result from reduced sleep.

We previously identified that the targeted expression of a CREB repressor in MB α/β neurons during the memory maintenance phase (48 to 72 hours after conditioning) impairs LTM [9], indicating that CREB-dependent transcription in MB α/β neurons is necessary for the maintenance of courtship LTM. Similarly, in this study, our results support the idea that Ap/Chi-dependent transcription in MB α/β neurons contributes to LTM maintenance for 2 days or longer. Taken together, courtship LTM is likely stored, at least in part, in MB α/β neurons from the second day after conditioning, and proteins required for maintaining LTM for 2 days or longer are possibly provided via transcriptions controlled by CREB and Ap/Chi. Although transcriptional regulations by CREB and Ap/Chi remain elusive, it will be of interest to investigate how the transcription by CREB and that by Ap/Chi are related and whether they occur in MB α/β neurons sequentially or in parallel.

The Pdf neuropeptide is necessary for the maintenance of courtship LTM, and the electrical silencing of Pdf neurons using Kir2.1 impairs LTM maintenance [9]. Furthermore, we found that the electrical silencing of Pdf neurons impairs memory consolidation (Fig 8E). Therefore, the electrical activity of Pdf neurons is essential for both memory consolidation and LTM maintenance. However, we found that the disruption of the Dynamin function using *shi*^*ts1*^ in Pdf neurons impairs memory consolidation but not LTM maintenance (Fig 8). These seemingly paradoxical consequences may result from the characteristic difference between the effects of Kir2.1 and mutated Dynamin (Shi^ts1^) on neurotransmission. The expression of *shi*^*ts1*^ in target neurons leads to the temperature-dependent blockage of synaptic vesicle recycling and thus synaptic neurotransmission [42]. On the other hand, the expression of *Kir2*.*1* in target neurons suppresses the excitability of the target neurons. Consequently, neurotransmission from the target neurons should be blocked [53]. Although the Pdf neuropeptide is essential for the generation of locomotor activity rhythms in *Drosophila* [54], the disruption of the Dynamin function in Pdf neurons has little effect on locomotor activity rhythms [55]. However, the temporal induction of Kir2.1 in Pdf neurons can induce arrhythmic locomotor activity [56]. On the basis of these findings, it is considered that Kir2.1 blocks Pdf release, but Dynamin-dependent synaptic transmission has little effect on Pdf release. We previously identified by qRT-PCR analysis that Pdf neuron–specific Ap knockdown does not affect Pdf expression [24]. Furthermore, Pdf knockdown impairs LTM maintenance; nevertheless, it does not affect memory consolidation [9]. Considering these findings, it is unlikely that Pdf release from l-LNvs plays a significant role in memory consolidation. Overall, our findings raise the possibility that Dynamin-dependent synaptic transmission (e.g., classical neurotransmitter release) from l-LNvs contributes to memory consolidation to establish courtship LTM.

In *Drosophila*, the LTM maintenance phase has been conceptually defined as the time after LTM is fully formed and consolidated, and it is generally believed that memory consolidation is completed within 1 day after conditioning [1,2]. Hirano and colleagues have reported that the consolidation and maintenance phases are molecularly separated in aversive olfactory LTM [10]. Similarly, we have proposed that the maintenance phase in courtship LTM is also molecularly distinguished from the memory consolidation phase [9]. Because *Pdf*^*01*^ mutant flies display intact 1-day memory but impaired 2-day memory [9], it is possible that the maintenance phase can be distinguished from the consolidation phase on the basis of the requirement of the Pdf function. In this study, we found that *ap*^*null*^/*Pdf*-GAL4; UAS-*ap*/+ flies also show intact 1-day memory but impaired 2-day memory (Fig 5), suggesting that the consolidation and maintenance phases in courtship LTM are molecularly and cellularly separated.

Ap is evolutionarily conserved in vertebrates and invertebrates, and its function in regulating developmental events is also well conserved [17]. Lhx2, a mammalian ortholog of Ap, plays essential roles in neurodevelopmental events (e.g., cell proliferation, axon pathfinding, and neurite outgrowth) in the central nervous system [17,57,58]. In particular, Lhx2 is involved in the development of the mouse hippocampus, which is one of the crucial brain structures regulating learning and memory [57,59–62]. As was observed in *Drosophila* Ap, Lhx2 also continues to be expressed in the mature hippocampus [63], suggesting its role in hippocampus-dependent brain functions such as learning and memory. Although little is known about the functional significance of mammalian Ap orthologues in the adult brain, our findings raise the interesting possibility that they are also involved in memory processes.

## Materials and methods

### Fly stocks

All flies were raised on glucose–yeast–cornmeal medium in 12:12 LD cycles at 25.0 ± 0.5°C (45% to 60% relative humidity). Virgin males and females were collected without anesthesia within 8 hours after eclosion. The fly stocks used for this study were as follows: WT Canton-S (CS), *ap*^*rk568*^ [Bloomington stock number (BL) 5374)], *ap*^*UGO35*^ (provided by Dr. Manfred Frasch, University of Erlangen-Nuremberg), *ap*^*md544*^ (BL3041), *ap*::*GFP* (BL38423), *Chi*^*e5*.*5*^ (Kyoto 107789), *Pdf*-GAL4 (BL6900), *c929* (BL25373), *R18F07* (BL47876), *R14F03* (BL69564), *R61G12* (BL41286), *R61G12*-LexA (BL52685), *R41C10* (BL50121), *R55D03* (BL47656), *c305a* (BL30829), *nSyb*-GAL4 (BL51635), hs-GAL4 [31], *OK107* [31], *30Y* [31], MB-LexA [64], UAS-*shi*^*ts1*^ [42], UAS-*Kir2*.*1*::*eGFP* (BL6596), UAS-*mCD8*::*GFP* (BL5137), UAS-*mCherry*::*NLS* (BL38424), UAS-*ap* RNAi (NIG-fly, 8376R-1), UAS-*ap* RNAi-TRiP (BL41673), UAS-*ap* (see next section), UAS-*Chi* RNAi (VDRC, 30454), UAS-*Chi* RNAi-TRiP (BL35435), UAS-*Rdl* (BL29036), UAS-*Rdl* RNAi (VDRC, 41103), UAS-*SuperClomeleon* (BL59847), LexAop-*SuperClomeleon* (BL59846), *tub*-GAL80^ts^ (BL7017), LexAop2-*FLPL* (BL55820), UAS>stop>*mCD8*::*GFP* (BL30032), and UAS>stop>*ap* RNAi (see next section). All lines used for behavior experiments except for *ap*^*UGO35*^, *Chi*^*e5*.*5*^, UAS-*mCD8*::*GFP*, and UAS>stop>*mCD8*::*GFP* were outcrossed for at least 5 generations to *white*^*1118*^ flies with the CS genetic background.

### Generation of transgenic flies

Full-length *ap* cDNA was synthesized by RT-PCR with adult fly head RNA and 2 primers, 5′-GCGGCCGCCAAAATGGGCGTCTGCACCGAGGAGCGC-3′ and 5′-TCTAGATTAGTCCAAGTTAAGTGGCGGTGTGC-3′. The PCR product was digested with NotI and XbaI, and cloned into a pBluescript (pBS) II SK(+). The *ap* cDNA was subcloned into the NotI/XbaI-digested pUAST attB vector [65]. Constructs of UAS-*ap* were injected into eggs of PBac{y[+]-attP-9A VK00005 (BL24862).

For the generation of the UAS-FRT-stop-FRT*-ap* RNAi construct (UAS*>*stop*>ap* RNAi), an *ap* RANi fragment was obtained by PCR from the genomic DNA of UAS-*ap* RNAi (NIG-fly, 8376R-1). Subsequently, it was inserted into the pUAST attB vector using an In-Fusion HD Cloning Kit (Takara Bio, Japan). The primer sequences used for the PCR are as follows: forward, 5′-AACAGATCTGCGGCCGCATAACGCGCAACCTCGAC-3′; reverse, 5′-ACAAAGATCCTCTAGAGGAACAATGCTCCGACTAG-3′. Next, the FRT-stop-FRT cassette (<stop<) was PCR-amplified from the UAS<stop<*mCD8*::*GFP* vector (Addgene, 24385) and then cloned into the pUAST attB vector containing the *ap* RNAi fragment by using an In-Fusion HD Cloning Kit. The primer sequences used for the PCR are as follows: forward, 5′-AGGGAATTGGGAATTCGAAGTTCCTATTCCGAAG-3′; reverse, 5′-ATCTGTTAACGAATTCGAAGTTCCTATACTTTCTAG-3′. The UAS>stop>*ap* RNAi construct was also injected into eggs of PBac{y[+]-attP-9A}VK00005.

### Courtship conditioning assay

The courtship conditioning assay was carried out as previously described [9]. For LTM, a 3- to 5-day-old male was placed with a mated female (4 to 7 day olds) in a conditioning chamber (15-mm diameter, 5-mm depth) containing food for 7 hours either with (conditioned) or without (naive) a single premated female (7-hour conditioning). If males remated with mated females during conditioning, we discarded such males after conditioning. After 7-hour conditioning, only flies showing courtship behaviors toward the mated female but not copulating successfully were transferred to a glass tube with food (12-mm diameter × 75-mm depth) and kept in isolation for 1, 2, or 5 days until the test. For STM, a 3- to 5-day-old male was placed with a mated female (4 to 7 day olds) in a conditioning chamber (15-mm diameter, 5-mm depth) without food for 1 hour either with (conditioned) or without (naive) a single premated female (1-hour conditioning). After 1-hour conditioning, only flies showing courtship behaviors toward the mated female but not copulating successfully were transferred to a glass tube with food (12 mm diameter × 75 mm depth) and kept in isolation for 1, 8, or 24 hours until the test.

The test was performed using a freeze-killed virgin female in a test chamber (15-mm diameter, 3-mm depth). All procedures in the experiments were carried out at 25 ± 1.0°C (45% to 60% relative humidity) except for the temperature shift experiments. The male courtship activity of individual flies was evaluated as a CI, defined as the percentage of time spent in performing courtship behaviors during a 10-minute observation period. We first measured the CI valiues of conditioned and naive males (CI_Conditioned_ and CI_Naive_), and then mean CI_Naive_ was calculated. To quantify courtship memory, the MI was calculated using the following formula: MI = (mean CI_Naive_ − mean CI_Conditioned_) /mean CI_Naive_.

### Heat-shock treatment

In F_1_ males between hs-GAL4 and UAS-*ap* RNAi flies, the *ap* expression was inhibited after heat-shock treatment. For qRT-PCR analysis, hs-GAL4/+, UAS-*ap* RNAi/+, and hs-GAL4/ UAS-*ap* RNAi flies were heat-shocked (37°C) for 20 minutes, and total RNA was extracted 3 hours after heat-shock treatment. For courtship conditioning assay, hs-GAL4/UAS-*ap* RNAi flies were heat-shocked 3 hours before conditioning, immediately after conditioning, or 3 hours before the test.

### Temporal disruption of neurotransmission

The temperature-sensitive allele *shibire*^*ts1*^ (*shi*^*ts1*^) is defective in Dynamin function at an RT. In F_1_ males between *Pdf*-GAL4 and UAS-*shi*^*ts1*^, *shi*^*ts1*^ is expressed in Pdf neurons. In these males, neurotransmission in Pdf neurons should be disrupted at RT but not at a PT (25°C). To disrupt neurotransmission in Pdf neurons during the memory consolidation, maintenance, or test phase, the temperature was increased to RT (32°C) during 3 experimental periods: conditioning phase, 48 to 72 hours after conditioning (memory maintenance phase), and 12 hours before the test initiation.

### Temporal gene expression using the TARGET system

The *tub*-GAL80^ts^ transgene used in the TARGET system [35] encodes a ubiquitously expressed and temperature-sensitive GAL4 repressor that is active at PT (25°C) but not at RT (30°C). By using UAS-*ap* RNAi or UAS-*Chi* RNAi combined with the TARGET system, we knocked down *ap* in GAL4-positive neurons at RT, but not at PT. We shifted PT to RT and vice versa during 2 experimental phases: 24 hours before the end of conditioning and 48 to 72 hours after conditioning. To drive the expression of *Kir2*.*1* in Pdf neurons during memory consolidation, flies were kept at RT during conditioning.

### *ap* knockdown in large ventral lateral clock neurons

Pdf neurons are classified into 2 neural clusters, s-LNvs and l-LNvs. To assay whether l-LNv–specific *ap* knockdown affects LTM, 2 binary gene expression systems (GAL4/UAS and LexA/LexAop) combined with Flippase (FLP/FRT) were used. The specific target gene is expressed in GAL4- and LexA-coexpressing neurons utilizing this system. *R61G12*-LexA, *c929*, and UAS*>*stop*>ap* RNAi lines were used in the experiments.

### Real-time quantitative reverse transcription PCR

For *ap*, TRizol (Invitrogen, USA) was used for collecting total RNA from about 30 male fly heads in each genotype. For *Chi*, a PicoPure RNA Isolation Kit (KIT0204, Thermo Fisher Scientific, USA) was used for collecting total RNA from 3 whole brains in each genotype. cDNA was synthesized by the reverse transcription reaction using a QuantiTect Reverse Transcription Kit (#205311, QIAGEN, USA). qRT-PCR was carried out using a Chromo 4 detector (CFB-3240, MJ Research) and the SYBR Premix Ex Taq (Takara Bio, Japan) for *ap* or the THUNDERBIRD SYBR qPCR Mix (QPS-201, TOYOBO) for *Chi*. The primer sequences used for qRT-PCR were as follows: *ap*-Forward, 5′- ATAACGCGCAACCTCGACGAC-3′; *ap*-Reverse, 5′- CATGAGGATTCCCGTTCCAGC-3′; *Chi*-Forward, 5′- AACGGGCCGTGAAAAGTGTG-3′; *Chi*-Reverse, 5′- GTGGTCGGTTCTATCGGGCA -3′; *rp49*-Forward, 5′-AAGATCGTGAAGAAGCGCAC-3′; *rp49*-Reverse, 5′-TGTGCACCAGGAACTTCTTG-3′. The expression level of each mRNA was normalized to that of *rp49* mRNA. The average of the normalized mRNA expression levels in control flies was calculated using data from 5 or 6 independent assays.

### Immunohistochemistry

Immunohistochemistry was performed as previously described [24]. For Pdf staining, brains were stained with a mouse anti-Pdf antibody (PDF C7-s, Developmental Studies Hybridoma Bank at the University of Iowa, 1:200) followed by Alexa Fluor 568 anti-mouse IgG (A11004, Thermo Fisher Scientific) as the secondary antibody (1:1,000). For GFP staining, brains were stained with a rabbit anti-GFP antibody (A11122, Thermo Fisher Scientific, 1:200), followed by Alexa Fluor 488 anti-rabbit IgG (A11008, Thermo Fisher Scientific, 1:1,000) as the secondary antibody. Fluorescence signals were observed under a confocal microscope [(LSM710, Zeiss, Germany) or (C2, Nikon, Japan)].

### Functional fluorescence imaging

Brains were prepared for imaging analysis as previously described with some modifications [66]. Briefly, brains were extracted in ice-cold Ca^2+^-free HL3 medium (in mM, NaCl, 70; sucrose, 115; KCl, 5; MgCl_2_, 20; NaHCO_3_, 10; trehalose, 5; Hepes, 5) [67]. Phenol red was used as a pH indicator (final concentration, 0.5 mg/ml) to adjust the pH of buffers by coloring. For the adjustment of pH, CO_2_ gas was dissolved in buffers so that the color became orange (pH 7.0 ± 0.2). The brains were treated with papain (10 U/ml, activated by 15-minute incubation with 0.8 mM EDTA at 37°C) for 15 minutes at RT and then washed several times with Ca^2+^-free HL3 medium. After papain treatment, the brains were incubated with standard *Drosophila* HL3 medium (in mM, CaCl_2_, 1.8; NaCl, 70; sucrose, 115; KCl, 5; MgCl_2_, 20; NaHCO_3_, 10; trehalose, 5; Hepes, 5) with TTX alone (1 μM) or with TTX (1 μM) and PTX (100 μM). During incubation, fresh medium was infused into the chamber using a peristaltic pump (AC-2110, ATTO, Japan) every 5 to 10 minutes.

For Cl^−^ imaging using SuperClomeleon, fluorescence images were captured at 0.5 Hz at 5-second intervals using a confocal microscopy system (C2, Nikon) equipped with a 20× dry objective (NA, 0.75; Nikon). The cyan fluorescent protein (CFP) was excited at 458 nm and detected using a 482 ± 17.5 nm band-pass filter, and the yellow fluorescent protein (YFP) was detected simultaneously using a 540 ± 15 nm band-pass filter. GABA was diluted in the incubation medium so that the final concentration became 400 μM, and 1.6 ml of the GABA solution was perfused 90 seconds after the start of capturing images. To calculate fluorescence resonance energy transfer (FRET) changes, the following analysis was performed using custom-made software developed in MATLAB (MathWorks, Japan). For the Cl^−^ imaging of l-LNvs, regions of interest (ROIs) were selected as a circle within or surrounding each l-LNv soma to measure the average F of CFP and YFP at each time point. For the Cl^−^ imaging of MB α/β neurons, ROIs were selected as a circle within each α lobe tip. We calculated FRET changes as R using the following formula: R = average F of CFP/average F of YFP. We calculated the initial R (R_0_) by averaging the R values recorded from 10 sequential frames before stimulation. To obtain ΔR/R_0_ (%) as Cl^−^ responses, we calculated (R–R_0_)/R_0_ × 100 at each time point.

### Statistical analyses

All the statistical analyses were performed using IBM SPSS Statistics 22 (IBM Japan) or BellCurve for Excel (Social Survey Research Information, Japan), except for the comparisons of MI. In all statistical analyses except for the comparisons of MI, the Kolmogorov–Smirnov test was used to determine whether the data are normally distributed. In the statistical analysis of CI, when basic data were not distributed, normally, we carried out the log transformation of the data. Even though the basic data or transformed data are normally distributed, heteroscedasticity was detected in all data. Thus, we used the Mann–Whitney *U* test for comparisons. In the statistical analysis of MI, the permutation test with 10,000 random permutations was used (H_0_, the difference between experimental and control groups is 0). The free statistical package R was used for these tests [68]. In the qRT-PCR of *ap* knockdown, Student *t* test or the Mann–Whitney *U* test was used for comparisons between HS− and HS+. In the qRT-PCR of *Chi* knockdown, 1-way ANOVA followed by post hoc analysis using Scheffe test was used for multiple comparisons. In functional fluorescence imaging, the basic data related to picrotoxin treatment were distributed normally and homoscedasticity was evident. Thus, Student *t* test was carried out. In the Cl^−^ imaging of l-LNvs, heteroscedasticity was evident in multiple comparisons among 3 genotypes (WT, *ap*^*nill*^/+, and *Chi*^*null*^/+ flies). Therefore, nonparametric ANOVA (Kruskal–Wallis test) followed by post hoc analysis using the Steel–Dwass test was carried out. In the Cl^−^ imaging of MB α lobes, the log-transformed data were distributed normally, and homoscedasticity was evident. Thus, 1-way ANOVA followed by post hoc analysis using Scheffe test was carried out. The numerical data used in all figures are included in S1 Data.

## Supporting information

S1 Data Excel spreadsheet containing the underlying numerical data for Figs 1, 2A, 3, 4A, 4B, 4D, 5A, 5B, 5D, 6, 7, 8 and 9 and S1, S2, S3, S4, S5, S6C, S6D and S8 Figs. (XLSX)

S1 Fig STM in WT and *ap*^*rk568*^/+ flies lasts for 8 hours but disappears 24 hours after conditioning. **(A)** WT flies were used in the experiments. Males were tested 8 hours and 24 hours after 1-hour conditioning. **(B)** *ap*^*rk568*^/+ males were tested 8 hours and 24 hours after 1-hour conditioning. (A and B) Box plots for a set of CI data show fifth, 25th, 75th, and 95th centiles. In the box and whisker plots, the black square in each box indicates the mean, the line in each box is drawn at the median, the white boxes indicate naive males, and the gray boxes indicate conditioned males. The underlying data can be found in S1 Data. CI, courtship index; MI, memory index; *N*, sample size; *U*, Mann–Whitney *U*; *P*, probability; *, *P* < 0.05; NS, not significant. Ap, Apterous; STM, short-term memory; WT, wild-type.
(TIF)

S2 Fig Real-time qRT-PCR analysis of *ap* mRNA expression level 48 hours after heat-shock treatment. HS−, non–heat-shocked flies. HS+, flies with heat-shock treatment (20 minutes) 48 hours before RNA extraction. NS, not significant. *N* = 3 to 5 in each bar. Error bars show SEM in each figure. The underlying data can be found in S1 Data. Ap, Apterous; qRT-PCR, quantitative reverse transcription PCR.
(TIF)

S3 Fig Heat-shock treatment does not affect LTM in control flies. **(A)** UAS-*ap* RNAi/+ flies were used. Five-day memory after 7-hour conditioning. NS, not significant. **(B)** hs-GAL4/+ flies were used. Five-day memory after 7-hour conditioning. (A and B) Box plots for a set of CI data show fifth, 25th, 75th, and 95th centiles. In the box and whisker plots, the black square in each box indicates the mean, the line in each box is drawn at the median, the white boxes indicate naive males, and the gray boxes indicate conditioned males. The underlying data can be found in S1 Data. CI, courtship index; MI, memory index; *N*, sample size; *U*, Mann–Whitney *U*; *P*, probability; NS, not significant. Ap, Apterous; LTM, long-term memory; RNAi, RNA interference.
(TIF)

S4 Fig Pan-neural knockdown of *ap* or *Chi* impairs LTM. **(A)** Pan-neuronal knockdown of *ap*. UAS-*ap* RNAi (NIG-fly) and UAS-*ap* RNAi (TRiP) lines were used. **(B)** Pan-neuronal knockdown of *Chi*. UAS-*Chi* RNAi (VDRC) and UAS-*Chi* RNAi (TRiP) lines were used. (A and B) *nSyb*-GAL4 was used as a pan-neuronal GAL4 line. *nSyb*-GAL4/UAS-*mCD8*::*GFP* flies were used as a control. Box plots for a set of CI data show fifth, 25th, 75th, and 95th centiles. In the box and whisker plots, the black square in each box indicates the mean, the line in each box is drawn at the median, the white boxes indicate naive males, and the gray boxes indicate conditioned males. The underlying data can be found in S1 Data. CI, courtship index; MI, memory index; *N*, sample size; *U*, Mann–Whitney *U*; *P*, probability; ***, *P* < 0.001. Ap, Apterous; Chi, Chip; LTM, long-term memory; RNAi, RNA interference.
(TIF)

S5 Fig Induction of *ap* RNAi by MB-GAL4 lines impairs LTM. **(A)** *OK107* was used. **, *P* < 0.01; NS, not significant. **(B)** *30Y* was used. *, *P* < 0.05; NS, not significant. (A and B) Five-day memory after 7-hour conditioning. Box plots for a set of CI data show fifth, 25th, 75th, and 95th centiles. In the box and whisker plots, the black square in each box indicates the mean, the line in each box is drawn at the median, the white boxes indicate naive males, and the gray boxes indicate conditioned males. The underlying data can be found in S1 Data. CI, courtship index; MI, memory index; *N*, sample size; *U*, Mann–Whitney *U*; *P*, probability. *, *P* < 0.05; **, *P* < 0.01; NS, not significant. Ap, Apterous; LTM, long-term memory; MB, mushroom body; RNAi, RNA interference.
(TIF)

S6 Fig Phenotypic analysis of *ap*^*GAL4*^. **(A)** Stacked confocal images showing an anterior view of the adult brain. Scale bars represent 100 μm. *ap*^*GAL4*^*/+*; UAS-*mCD8*::*GFP* flies were used. **(B)** Stacked confocal images showing an anterior view of the adult brain. Scale bars represent 100 μm. *ap*^*GAL4*^*/*MB-GAL80; UAS-*mCD8*::*GFP* flies were used. (A and B) Green, mCD8::GFP; Magenta, Fas II. For Fas II staining, brains were stained with a mouse anti-Fas II antibody (1D4 anti-Fas II, Developmental Studies Hybridoma Bank at the University of Iowa, 1:500) followed by Alexa Fluor 568 anti-mouse IgG (A11004, Thermo Fisher Scientific) as the secondary antibody (1:1,000). **(C)** WT and *ap*^*rk568*^/+ flies were used in the experiments. Males were tested 1 hour after 1-hour conditioning (1-hour memory). **(D)** WT and *ap*^*GAL4*^/+ flies were used in the experiments. Males were tested on day 5 after 7-hour conditioning (5-day memory). (C and D) Box plots for a set of CI data show fifth, 25th, 75th, and 95th centiles. In the box and whisker plots, the black square in each box indicates the mean, the line in each box is drawn at the median, the white boxes indicate naive males, and the gray boxes indicate conditioned males. The underlying data can be found in S1 Data. CI, courtship index; MI, memory index; *N*, sample size; *U*, Mann–Whitney *U*; *P*, probability. *, *P* < 0.05; NS, not significant. Ap, Apterous; MB, mushroom body; WT, wild-type.
(TIF)

S7 Fig MB structure in *ap*^*GAL4*^/ *ap*^*rk568*^ and *ap*^*null*^/+ flies. **(A)** Stacked confocal image showing an anterior view of the adult brain. The scale bar represents 100 μm. *ap*^*GAL4*^*/ap*^*rk568*^; UAS-*mCD8*::*GFP* flies were used in the experiment. Arrows show MB α lobes. **(B)** Stacked confocal images of MB. Scale bars represent 50 μm. *ap*^*null*^*/+*; *R41C10*/UAS-*mCD8*::*GFP* and control (*R41C10*/UAS-*mCD8*::*GFP*) flies were used. Ap, Apterous; MB, mushroom body.
(TIF)

S8 Fig Ap in MB α/β neurons is necessary for the appropriate response to GABA. The underlying data can be found in S1 Data. (A and B) We used MB-LexA, LexAop-SuperClomeleon flies with both MB-LexA and LexAop-*SuperClomeleon* constructs in the second chromosome. **(A)** MB-LexA, LexAop-*SuperClomeleon*/+ flies (WT), MB-LexA, LexAop-*SuperClomeleon* /*ap*^*null*^ (*ap*^*nill*^/+), and MB-LexA, LexAop-*SuperClomeleon*/*Chi*^*null*^ (*Chi*^*null*^/+) flies were used. Traces of mean SuperClomeleon response to 3 mM GABA and 1 μM TTX. Error bars, SE; *N* = 11 to 14 in each trace. **(B)** Maximum percentage change in fluorescence of the SuperClomeleon related to (A). One-way ANOVA followed by post hoc analysis using Scheffe test was carried out for multiple comparisons. **(C)** Five-day memory after 7-hour conditioning in *R41C10*/UAS-*Rdl* flies. *R41C10*/UAS-*mCD8*::*GFP* flies were used as the control. NS, not significant. (B and C) Box plots show fifth, 25th, 75th, and 95th centiles. In the box and whisker plots, the black square in each box indicates the mean, and the line in each box is drawn at the median. NS, not significant. Ap, Apterous; GABA, gamma-aminobutyric acid; MB, mushroom body; WT, wild-type.
(TIF)

## Abbreviations

aMe: accessory medullae
Ap: Apterous
Bx: Beadex
CFP: cyan fluorescent protein
Chi: Chip
CI: courtship index
CREB: cAMP response element binding protein
CrebB: cyclic-AMP response element-binding protein B
dLMO: Drosophila LIM-only protein
FRET: fluorescence resonance energy transfer
GABA: gamma-aminobutyric acid
hs: heat-shock
LIM-HD: LIM homeodomain
l-LNv: large ventral–lateral clock neuron
LTM: long-term memory
MB: mushroom body
MI: memory index
Pdf: pigment dispersing factor
per: period
POT: posterior optic tract
PT: permissive temperature
qRT-PCR: quantitative reverse transcription PCR
Rdl: resistant to dieldrin
RNAi: RNA interference
ROI: region of interest
RT: restrictive temperature
s-LNv: small ventral–lateral neuron
STM: short-term memory
WT: wild-type
YFP: yellow fluorescent protein
