# Supplementary figures and images for: Consolidation and maintenance of long-term memory involve dual functions of the developmental regulator Apterous in clock neurons and mushroom bodies in the Drosophila brain
Source: PLoS Biol. 2021 Dec 3;19(12):e3001459. doi: 10.1371/journal.pbio.3001459 (PMC8641882; doi:10.1371/journal.pbio.3001459)

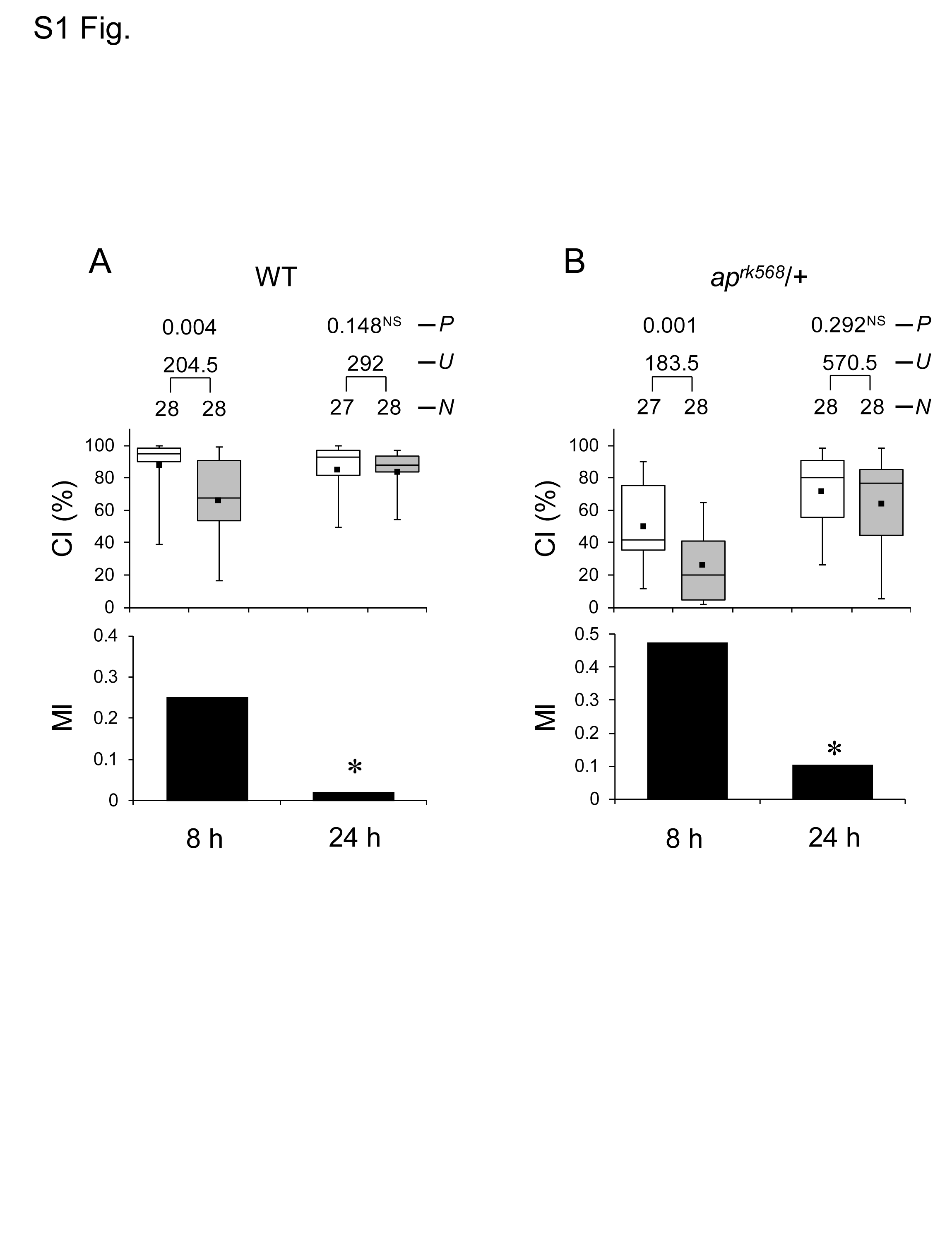

Supplement: S1 Fig — (A) WT flies were used in the experiments. Males were tested 8 hours and 24 hours after 1-hour conditioning. (B) aprk568/+ males were tested 8 hours and 24 hours after 1-hour conditioning. (A and B) Box plots for a set of CI data show fifth, 25th, 75th, and 95th centiles. In the box and whisker plots, the black square in each box indicates the mean, the line in each box is drawn at the median, the white boxes indicate naive males, and the gray boxes indicate conditioned males. The underlying data can be found in S1 Data. CI, courtship index; MI, memory index; N, sample size; U, Mann–Whitney U; P, probability; *, P < 0.05; NS, not significant. Ap, Apterous; STM, short-term memory; WT, wild-type. (TIF) [file pbio.3001459.s002.tif]

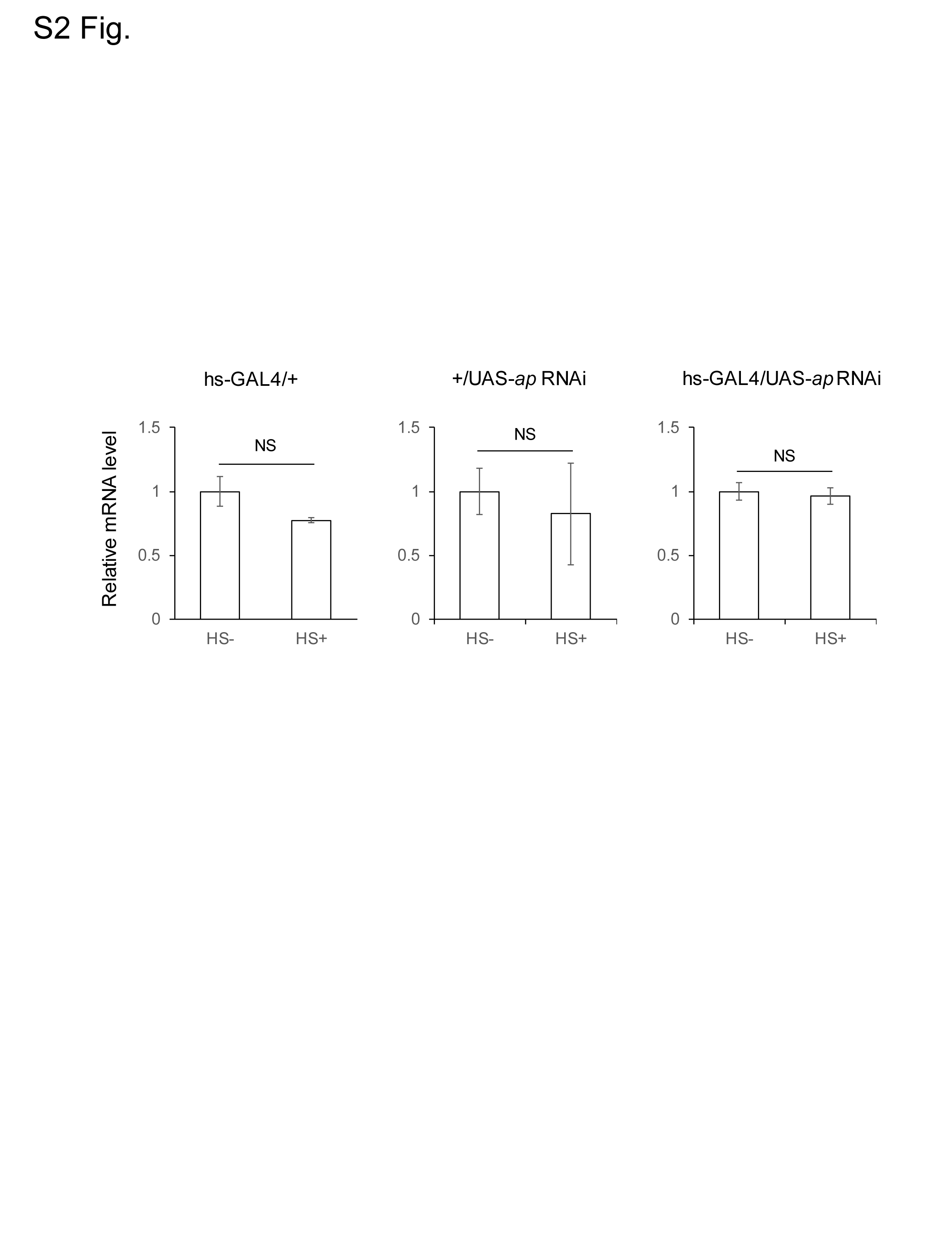

Supplement: S2 Fig — HS−, non–heat-shocked flies. HS+, flies with heat-shock treatment (20 minutes) 48 hours before RNA extraction. NS, not significant. N = 3 to 5 in each bar. Error bars show SEM in each figure. The underlying data can be found in S1 Data. Ap, Apterous; qRT-PCR, quantitative reverse transcription PCR. (TIF) [file pbio.3001459.s003.tif]

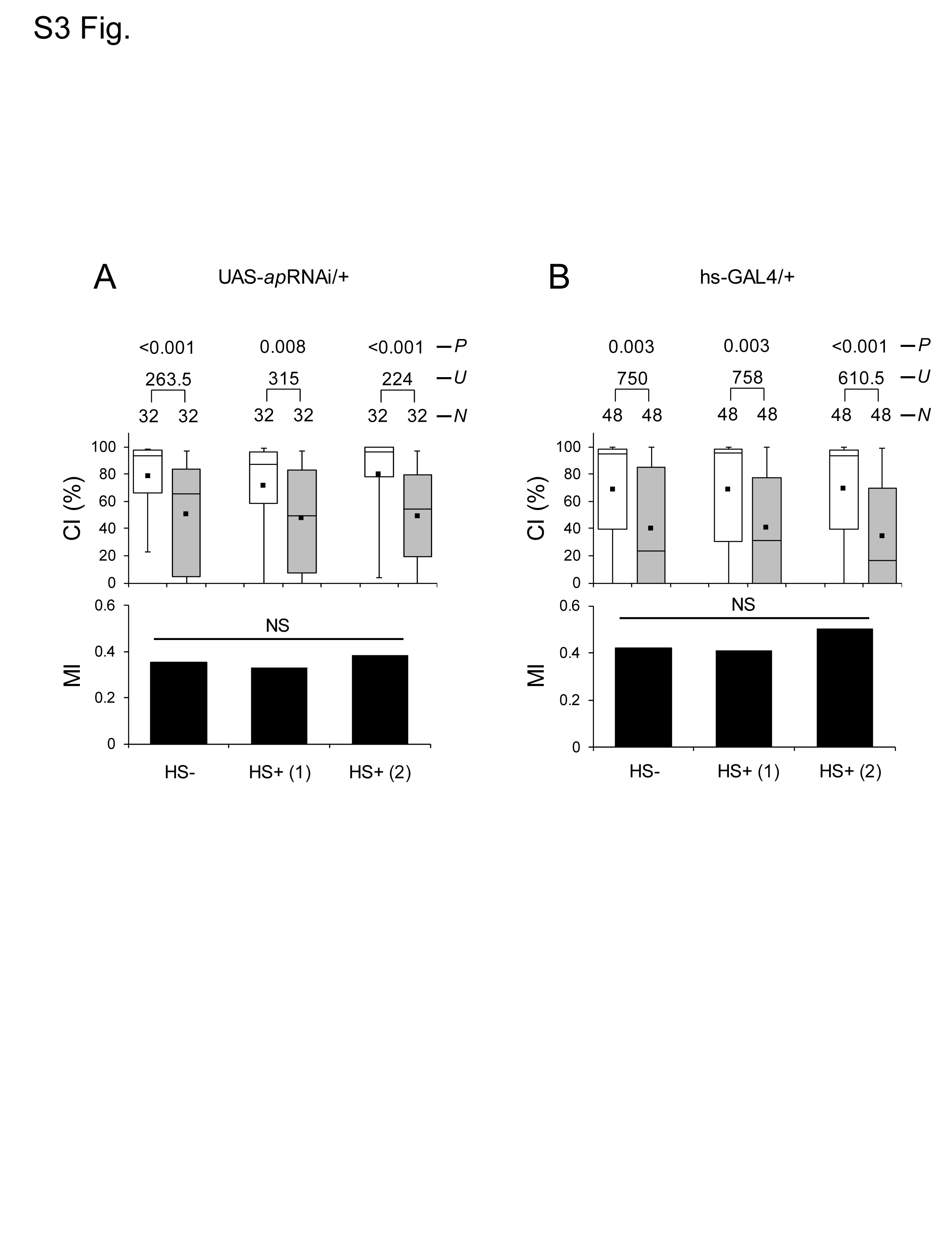

Supplement: S3 Fig — (A) UAS-ap RNAi/+ flies were used. Five-day memory after 7-hour conditioning. NS, not significant. (B) hs-GAL4/+ flies were used. Five-day memory after 7-hour conditioning. (A and B) Box plots for a set of CI data show fifth, 25th, 75th, and 95th centiles. In the box and whisker plots, the black square in each box indicates the mean, the line in each box is drawn at the median, the white boxes indicate naive males, and the gray boxes indicate conditioned males. The underlying data can be found in S1 Data. CI, courtship index; MI, memory index; N, sample size; U, Mann–Whitney U; P, probability; NS, not significant. Ap, Apterous; LTM, long-term memory; RNAi, RNA interference. (TIF) [file pbio.3001459.s004.tif]

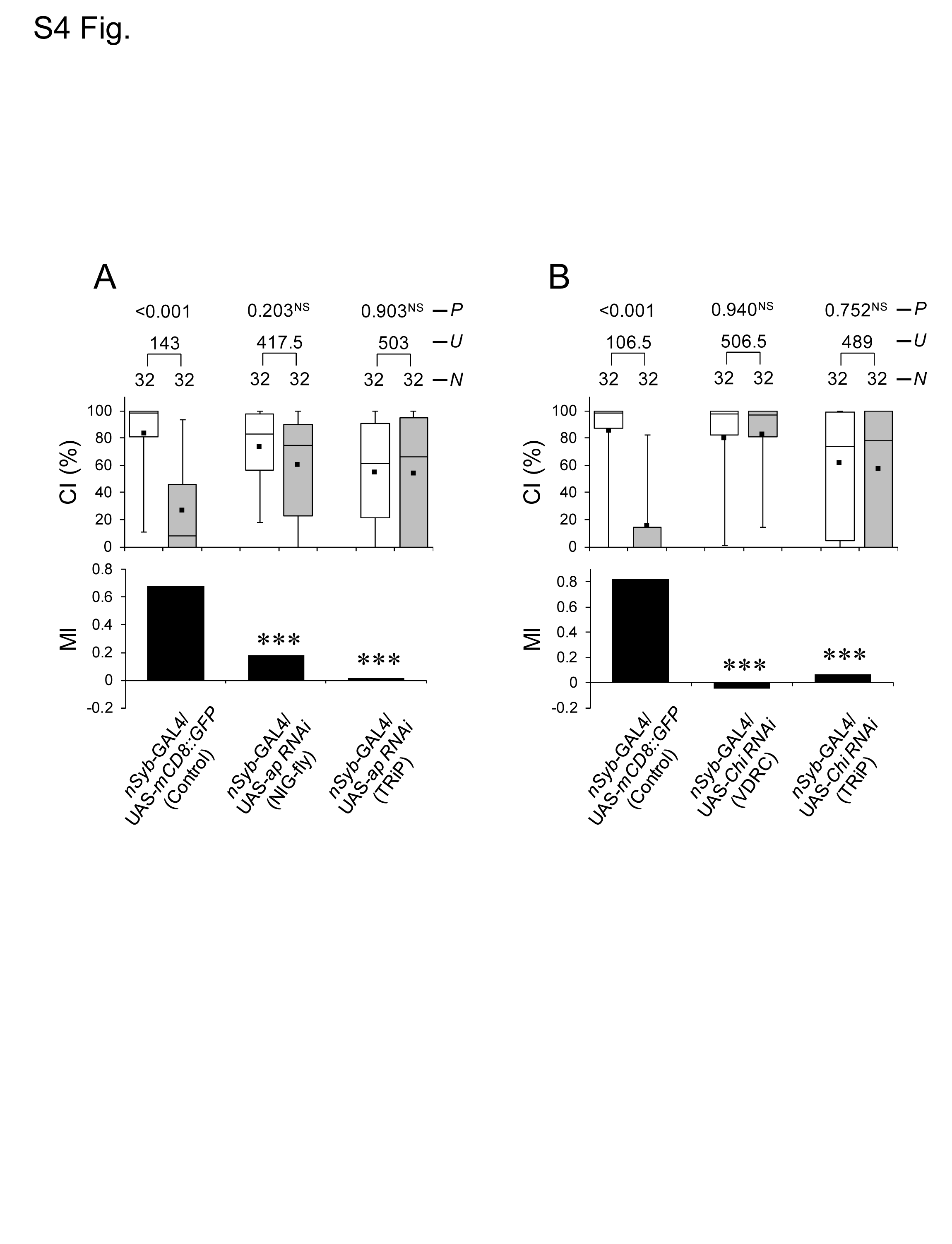

Supplement: S4 Fig — (A) Pan-neuronal knockdown of ap. UAS-ap RNAi (NIG-fly) and UAS-ap RNAi (TRiP) lines were used. (B) Pan-neuronal knockdown of Chi. UAS-Chi RNAi (VDRC) and UAS-Chi RNAi (TRiP) lines were used. (A and B) nSyb-GAL4 was used as a pan-neuronal GAL4 line. nSyb-GAL4/UAS-mCD8::GFP flies were used as a control. Box plots for a set of CI data show fifth, 25th, 75th, and 95th centiles. In the box and whisker plots, the black square in each box indicates the mean, the line in each box is drawn at the median, the white boxes indicate naive males, and the gray boxes indicate conditioned males. The underlying data can be found in S1 Data. CI, courtship index; MI, memory index; N, sample size; U, Mann–Whitney U; P, probability; ***, P < 0.001. Ap, Apterous; Chi, Chip; LTM, long-term memory; RNAi, RNA interference. (TIF) [file pbio.3001459.s005.tif]

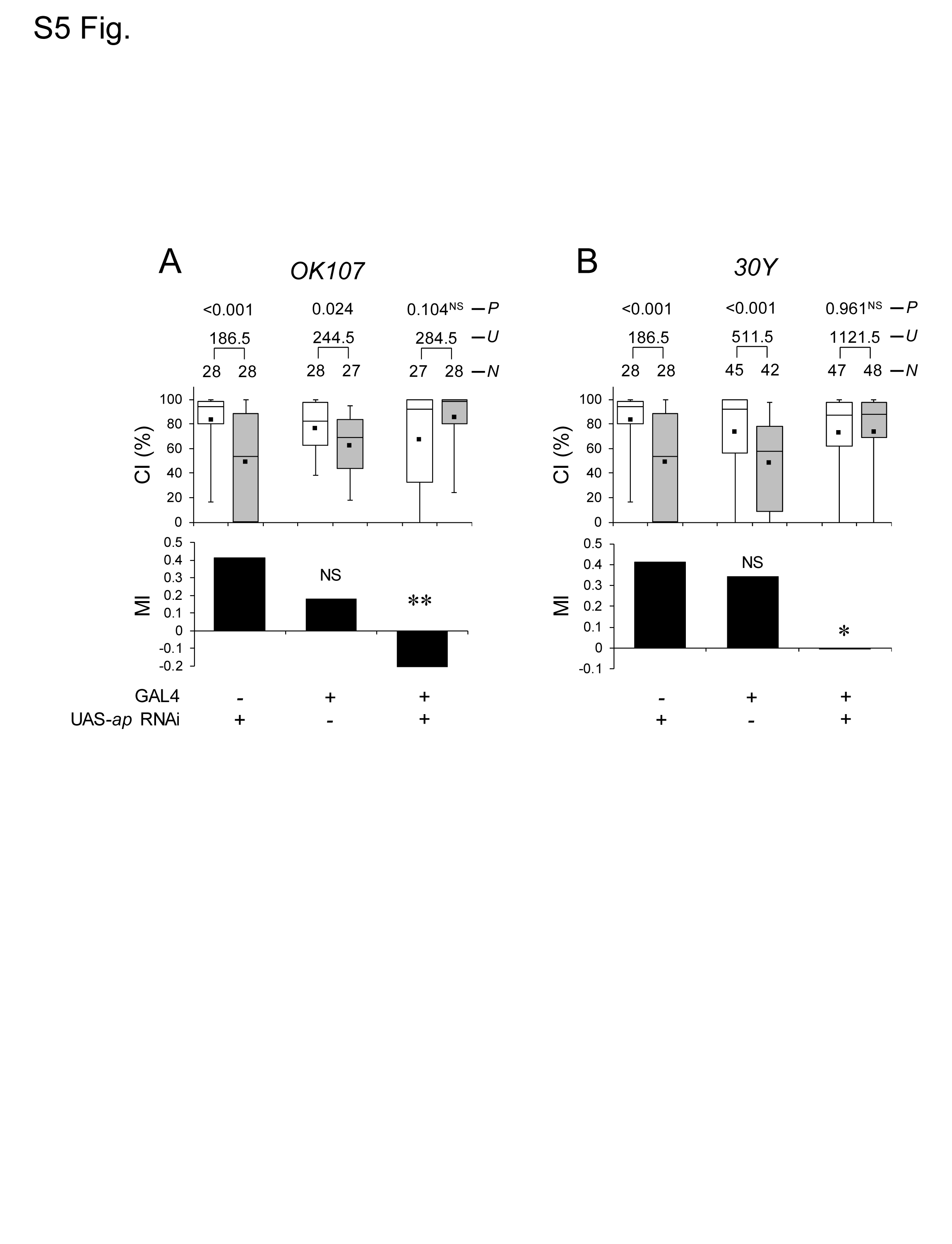

Supplement: S5 Fig — (A) OK107 was used. **, P < 0.01; NS, not significant. (B) 30Y was used. *, P < 0.05; NS, not significant. (A and B) Five-day memory after 7-hour conditioning. Box plots for a set of CI data show fifth, 25th, 75th, and 95th centiles. In the box and whisker plots, the black square in each box indicates the mean, the line in each box is drawn at the median, the white boxes indicate naive males, and the gray boxes indicate conditioned males. The underlying data can be found in S1 Data. CI, courtship index; MI, memory index; N, sample size; U, Mann–Whitney U; P, probability. *, P < 0.05; **, P < 0.01; NS, not significant. Ap, Apterous; LTM, long-term memory; MB, mushroom body; RNAi, RNA interference. (TIF) [file pbio.3001459.s006.tif]

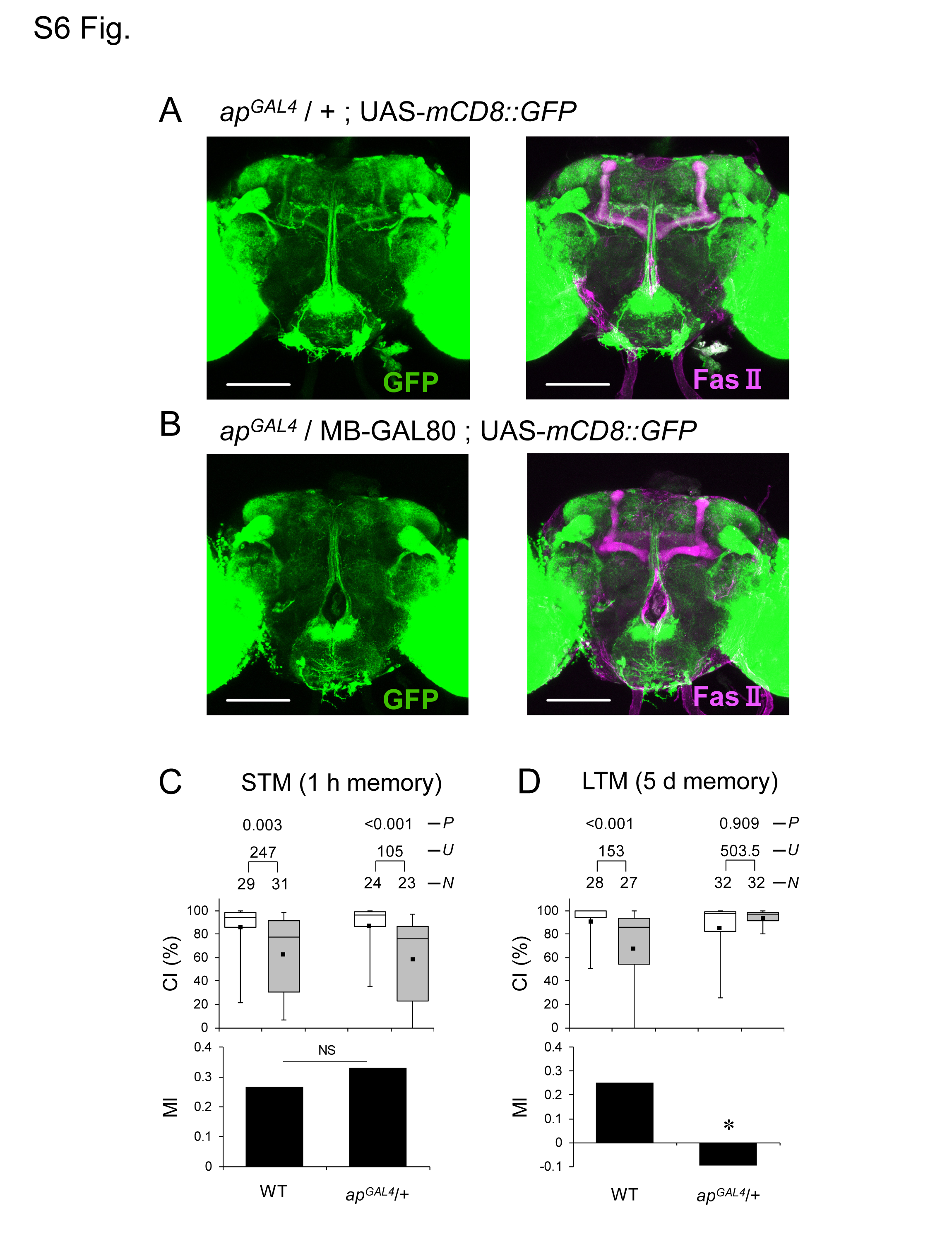

Supplement: S6 Fig — (A) Stacked confocal images showing an anterior view of the adult brain. Scale bars represent 100 μm. apGAL4/+; UAS-mCD8::GFP flies were used. (B) Stacked confocal images showing an anterior view of the adult brain. Scale bars represent 100 μm. apGAL4/MB-GAL80; UAS-mCD8::GFP flies were used. (A and B) Green, mCD8::GFP; Magenta, Fas II. For Fas II staining, brains were stained with a mouse anti-Fas II antibody (1D4 anti-Fas II, Developmental Studies Hybridoma Bank at the University of Iowa, 1:500) followed by Alexa Fluor 568 anti-mouse IgG (A11004, Thermo Fisher Scientific) as the secondary antibody (1:1,000). (C) WT and aprk568/+ flies were used in the experiments. Males were tested 1 hour after 1-hour conditioning (1-hour memory). (D) WT and apGAL4/+ flies were used in the experiments. Males were tested on day 5 after 7-hour conditioning (5-day memory). (C and D) Box plots for a set of CI data show fifth, 25th, 75th, and 95th centiles. In the box and whisker plots, the black square in each box indicates the mean, the line in each box is drawn at the median, the white boxes indicate naive males, and the gray boxes indicate conditioned males. The underlying data can be found in S1 Data. CI, courtship index; MI, memory index; N, sample size; U, Mann–Whitney U; P, probability. *, P < 0.05; NS, not significant. Ap, Apterous; MB, mushroom body; WT, wild-type. (TIF) [file pbio.3001459.s007.tif]

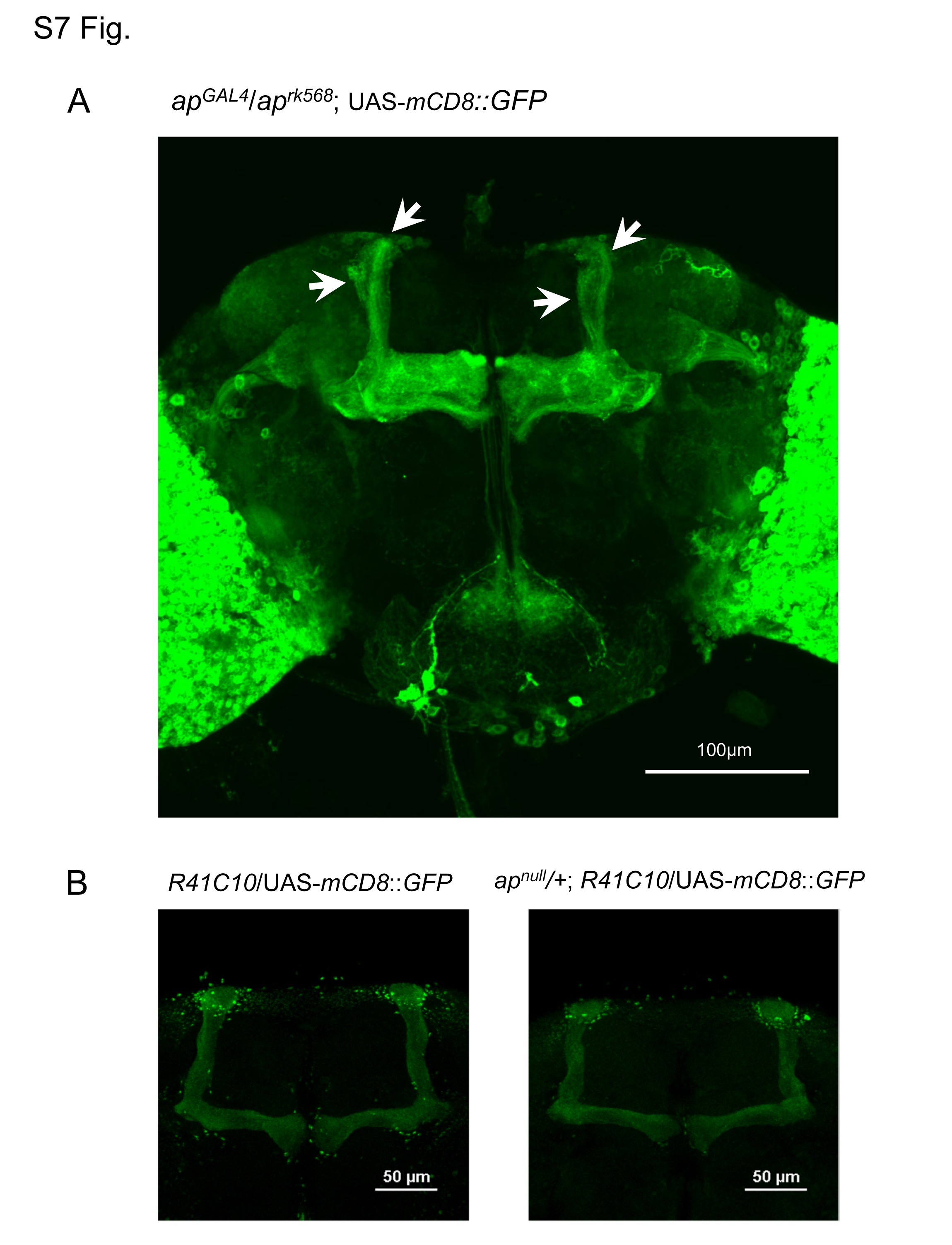

Supplement: S7 Fig — (A) Stacked confocal image showing an anterior view of the adult brain. The scale bar represents 100 μm. apGAL4/aprk568; UAS-mCD8::GFP flies were used in the experiment. Arrows show MB α lobes. (B) Stacked confocal images of MB. Scale bars represent 50 μm. apnull/+; R41C10/UAS-mCD8::GFP and control (R41C10/UAS-mCD8::GFP) flies were used. Ap, Apterous; MB, mushroom body. (TIF) [file pbio.3001459.s008.tif]

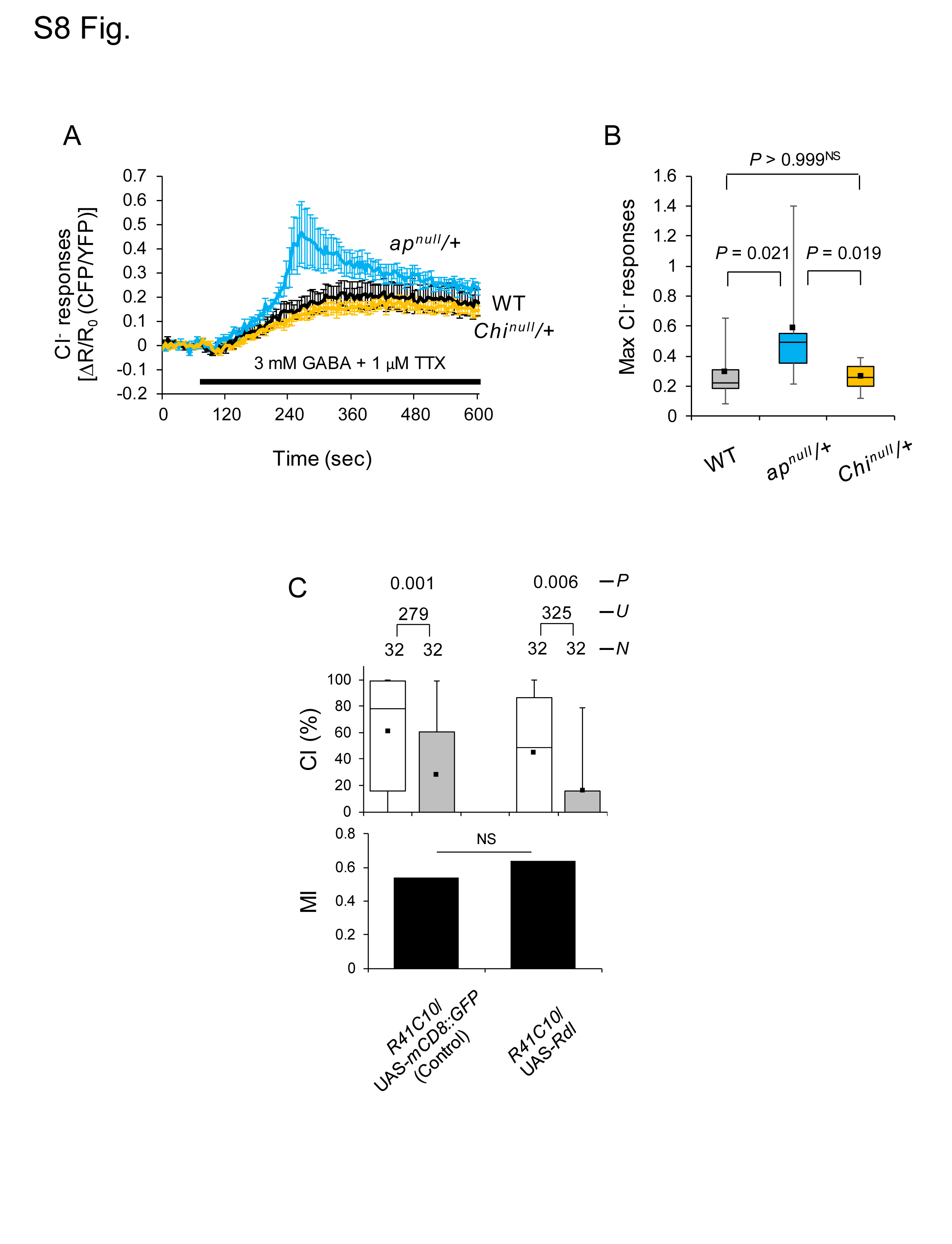

Supplement: S8 Fig — The underlying data can be found in S1 Data. (A and B) We used MB-LexA, LexAop-SuperClomeleon flies with both MB-LexA and LexAop-SuperClomeleon constructs in the second chromosome. (A) MB-LexA, LexAop-SuperClomeleon/+ flies (WT), MB-LexA, LexAop-SuperClomeleon /apnull (apnill/+), and MB-LexA, LexAop-SuperClomeleon/Chinull (Chinull/+) flies were used. Traces of mean SuperClomeleon response to 3 mM GABA and 1 μM TTX. Error bars, SE; N = 11 to 14 in each trace. (B) Maximum percentage change in fluorescence of the SuperClomeleon related to (A). One-way ANOVA followed by post hoc analysis using Scheffe test was carried out for multiple comparisons. (C) Five-day memory after 7-hour conditioning in R41C10/UAS-Rdl flies. R41C10/UAS-mCD8::GFP flies were used as the control. NS, not significant. (B and C) Box plots show fifth, 25th, 75th, and 95th centiles. In the box and whisker plots, the black square in each box indicates the mean, and the line in each box is drawn at the median. NS, not significant. Ap, Apterous; GABA, gamma-aminobutyric acid; MB, mushroom body; WT, wild-type. (TIF) [file pbio.3001459.s009.tif]
